# Sympatric or micro-allopatric speciation in a glacial lake? Genomic islands support neither

**DOI:** 10.1093/nsr/nwac291

**Published:** 2022-12-27

**Authors:** Ning Sun, Liandong Yang, Fei Tian, Honghui Zeng, Ziwen He, Kai Zhao, Cheng Wang, Minghui Meng, Chenguang Feng, Chengchi Fang, Wenqi Lv, Jing Bo, Yongtao Tang, Xiaoni Gan, Zuogang Peng, Yiyu Chen, Shunping He

**Affiliations:** State Key Laboratory of Freshwater Ecology and Biotechnology, Institute of Hydrobiology, Chinese Academy of Sciences, Wuhan 430072, China; University of Chinese Academy of Sciences, Beijing 100049, China; State Key Laboratory of Freshwater Ecology and Biotechnology, Institute of Hydrobiology, Chinese Academy of Sciences, Wuhan 430072, China; Key Laboratory of Adaptation and Evolution of Plateau Biota, Northwest Institute of Plateau Biology, Chinese Academy of Sciences, Xining 810008, China; State Key Laboratory of Freshwater Ecology and Biotechnology, Institute of Hydrobiology, Chinese Academy of Sciences, Wuhan 430072, China; State Key Laboratory of Biocontrol, Guangdong Key Lab of Plant Resources, School of Life Sciences, Sun Yat-sen University, Guangzhou 510275, China; Key Laboratory of Adaptation and Evolution of Plateau Biota, Northwest Institute of Plateau Biology, Chinese Academy of Sciences, Xining 810008, China; State Key Laboratory of Freshwater Ecology and Biotechnology, Institute of Hydrobiology, Chinese Academy of Sciences, Wuhan 430072, China; University of Chinese Academy of Sciences, Beijing 100049, China; State Key Laboratory of Freshwater Ecology and Biotechnology, Institute of Hydrobiology, Chinese Academy of Sciences, Wuhan 430072, China; State Key Laboratory of Freshwater Ecology and Biotechnology, Institute of Hydrobiology, Chinese Academy of Sciences, Wuhan 430072, China; School of Ecology and Environment, Northwestern Polytechnical University, Xi’an 710129, China; State Key Laboratory of Freshwater Ecology and Biotechnology, Institute of Hydrobiology, Chinese Academy of Sciences, Wuhan 430072, China; State Key Laboratory of Freshwater Ecology and Biotechnology, Institute of Hydrobiology, Chinese Academy of Sciences, Wuhan 430072, China; University of Chinese Academy of Sciences, Beijing 100049, China; Institute of Deep-Sea Science and Engineering, Chinese Academy of Sciences, Sanya 572000, China; University of Chinese Academy of Sciences, Beijing 100049, China; Key Laboratory of Adaptation and Evolution of Plateau Biota, Northwest Institute of Plateau Biology, Chinese Academy of Sciences, Xining 810008, China; State Key Laboratory of Freshwater Ecology and Biotechnology, Institute of Hydrobiology, Chinese Academy of Sciences, Wuhan 430072, China; Key Laboratory of Freshwater Fish Reproduction and Development (Ministry of Education), Southwest University School of Life Sciences, Chongqing 400700, China; National Natural Science Foundation of China, Beijing 100085, China; State Key Laboratory of Freshwater Ecology and Biotechnology, Institute of Hydrobiology, Chinese Academy of Sciences, Wuhan 430072, China; Institute of Deep-Sea Science and Engineering, Chinese Academy of Sciences, Sanya 572000, China; Center for Excellence in Animal Evolution and Genetics, Chinese Academy of Sciences, Kunming 650223, China

**Keywords:** sympatric speciation, gene flow, genomic islands, micro-parapatric speciation, selection, olfaction

## Abstract

Apparent cases of sympatric speciation may actually be due to micro-allopatric or micro-parapatric speciation. One way to distinguish between these models is to examine the existence and nature of genomic islands of divergence, wherein divergent DNA segments are interspersed with low-divergence segments. Such islands should be rare or absent under micro-allopatric speciation but common in cases of speciation with gene flow. Sympatric divergence of endemic fishes is known from isolated saline, crater, postglacial, and ancient lakes. Two morphologically distinct cyprinid fishes, *Gymnocypris eckloni scoliostomus* (GS) and *G. eckloni eckloni* (GE), in a small glacial lake on the Qinghai–Tibet Plateau, Lake Sunmcuo, match the biogeographic criteria of sympatric speciation. In this study, we examined genome-wide variation in 46 individuals from these two groups. The divergence time between the GS and GE lineages was estimated to be 20–60 Kya. We identified 54 large genomic islands (≥100 kb) of speciation, which accounted for 89.4% of the total length of all genomic islands. These islands harboured divergent genes related to olfactory receptors and olfaction signals that may play important roles in food selection and assortative mating in fishes. Although the genomic islands clearly indicated speciation with gene flow and rejected micro-allopatric speciation, they were too large to support the hypothesis of sympatric speciation. Theoretical and recent empirical studies suggested that continual gene flow in sympatry should give rise to many small genomic islands (as small as a few kilobases in size). Thus, the observed pattern is consistent with the extensive evidence on parapatric speciation, in which adjacent habitats facilitate divergent selection but also permit gene flow during speciation. We suggest that many, if not most, of the reported cases of sympatric speciation are likely to be micro-parapatric speciation.

## INTRODUCTION

Allopatric speciation requires geographic barriers that completely prevent gene flow and allow the populations to evolve independently, which eventually leads to reproductive isolation (RI) [1,2]. Sympatric speciation, proposed by Darwin [3], is the evolution of RI without geographic barriers, in which new species arise from a single ancestral population [4,5]. Some biogeographic criteria suggested that sympatric speciation can occur if the two species overlap in their cruising range [6]. However, this is merely a case of sympatric coexistence From a population genetic perspective, any case in which the spatial structure of progenitor populations affects habitat selection or mating is not considered pure sympatric speciation [7–9]. In this study, pure sympatric speciation was considered divergence within a single geographical region where the range of one nascent species completely overlaps the other. These diverging groups are not separated by any spatial structure barrier in the ancestral range. The initial gene exchange rate between This mode of speciation has been controversial for over a century, partly because antagonism between selection and recombination makes this mode of speciation theoretically difficult [8,10–12].

The key aspect of sympatric speciation is that incipient species can potentially exchange genes (genetic recombination) by interbreeding, which breaks up the correlation between co-adapted gene groups necessary for species formation [13]. Therefore, the potential for sympatric speciation may be greatly restricted unless there is strong disruptive selection [12,14–18]. This selection will cause the population to divide into two subpopulations, each specialized on a different resource. The hybrids between the subpopulations are poorly adapted to either resource and eventually suffer from reduced fitness [14]. Disruptive selection contributes to the fixation of locally adaptive alleles that are beneficial in their ecological niches or mate choice [19,20]. In this case, sympatric speciation can occur as a result of habitat isolation or sexual isolation [13].

With the advent of high-throughput sequencing, and the development and application of population genomic approaches, increasing numbers of empirical case studies on sympatric speciation in both plants and animals have been reassessed [21–38]. However, these approaches have limited ability to distinguish spatiotemporal overlap because almost all empirical case studies of sympatric speciation have some degree of spatiotemporal differentiation between sister taxa [21–38]. As a result, the most inclusive definition of sympatric will skew our understanding of the truth about the speciation process. For example, consider two sister species that diverged from a single population and occupied the same biogeographic range. They are considered sympatric in terms of their broad-scale biogeography. However, if speciation that results from specialization to different habitats within their range and genetic exchange is inherently non-random, it can be also argued that speciation was not truly sympatric but rather micro-allopatric [39] or micro-parapatric by fine-scale spatial partitioning. From a population genetic perspective, it is dubbed micro-allopatric [40] or micro-parapatric, if no gene flow or restricted gene flow occurs during some episodes before the completion of speciation. Consequently, it is difficult to distinguish sympatric, micro-allopatric, and micro-parapatric speciation based solely on geographical considerations.

**Figure 1.**
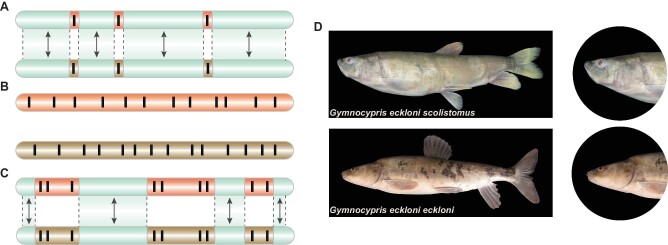
Gene flow modes under sympatry, allopatry and parapatry, and geographic locations of two *Gymnocypris* subspecies. The two horizontal bars represent the genomes of two diverging populations. (A) Sympatry: Bidirectional gene flow was frequent (+ +), and only a few loci (indicated by black lines) referred to as small genomic islands (−) are differentially adapted; genes at such loci are not exchanged between populations. Gene flow continues in the rest of the genome (arrows). Modified with permission from [41,42]. (B) Allopatry: There is no gene flow (−) between the two populations, and there is genome-wide differentiation. (C) Parapatry: The level of gene flow (+) between populations is lower in sympatry but higher than in allopatry. In this mode, adjacent habitats can facilitate divergent selection, which contributes to the formation of large genomic islands (+ +). Gene flow continues in the rest of the genome (arrows). (D) *Gymnocypris eckloni scoliostomus* and *G. eckloni eckloni* are morphologically distinct.

The main distinctions among the three modes are uninterrupted gene flow and the intensity of gene flow during speciation (Fig. 1A–C). In this genic view of speciation, speciation with gene flow has been shown to leave footprints in the form of genomic islands that are non-introgressable [27,41–45] (Fig. 1A–C). These genomic islands are frequently composed of highly divergent DNA regions interspersed with less divergent portions. In theory, the sizes of the genomic islands should be negatively correlated with gene flow between diverging populations. Therefore, sympatric speciation is expected to have small genomic islands owing to frequent bidirectional gene flow, whereas micro-parapatric speciation should leave footprints of large genomic islands, as in parapatric speciation, due to restricted gene flow. Micro-allopatric speciation will exhibit genome-wide differentiation, as in allopatric speciation, because geographic barriers prevented gene flow. In addition, these genomic islands may contain a small set of ‘speciation genes’ that govern ecological specialization or generate intrinsic genomic incompatibilities [46].

It has been suggested that the presence of endemic sister species in small circumscribed areas (e.g. isolated lakes or islands [13] or phytophagous insects) might indicate that these species originated sympatrically, such as *Howea forsteriana* and *H. belmoreana* on Lord Howe Island [47,48], cichlid fishes in crater lakes [22,27], and apple and hawthorn maggot flies [49–51]. These species are likely to undergo species differentiation in such habitats without apparent physical isolation. A previous study indicated that Lake Sunmcuo was a hydrographically isolated inland lake that did not flow into the Yellow River until approximately the Holocene period, and it is now connected by a torrential mountain stream [52]. This glacial lake is small (3.8 km^2^) [53], oligotrophic, and homogenous in habitat, all of which provide an ideal location for testing sympatric speciation [54].

*Gymnocypris eckloni scoliostomus* (GS) and *G. eckloni eckloni* (GE), two sister subspecies of *G. eckloni* (Cyprinidae: Schizothoracinae: *Gymnocypris*), are sympatrically distributed in Lake Sunmcuo in the Qinghai–Tibet Plateau, China at an altitude of 4100 m [55,56]. Despite their close relationship, these two subspecies show significant differences in morphology (Fig. 1D) and reproductive characteristics. GS has a terminal or superior mouth with a more oblique and deeply arched mouth cleft. It prefers to feed on plankton and spawns in July with salmon-pink eggs. By contrast, GE has a subterminal mouth without a horny ridge at the inner margin of the lower jaw. It feeds on plankton, zoobenthos, algae, hydrophytes, and small fish, and spawns in April and May with yellow eggs [52,54,55,57,58]. It is known that the shape of the fish's mouth and lower jaw are tightly linked to the different nutritional types and river depths of food. GS prefers to feed on the upper part of the water column or on shoal rich in plankton, whereas GE usually has a wider niche that corresponds to a variety of food items [46,59]. All these characteristics demonstrate that GS and GE satisfy the biogeographic criteria for sympatric speciation.

In this study, we constructed a highly contiguous genome assembly of GS, and surveyed genomic variation of the two species by whole-genome resequencing of 46 samples from Lake Sunmcuo to investigate the genomic patterns of divergence. We explored gene flow and identified highly divergent genome regions. Unexpectedly, we discovered many large genomic islands (≥100 kb) between GS and GE, even though continual gene flow in sympatry should give rise to many small genomic islands (a few kilobases in size). This pattern of genomic divergence is consistent with the extensive evidence on parapatric speciation, in which adjacent habitats facilitate divergent selection but also permit gene flow during speciation. This study will provide more accurate insights into sympatric speciation.

## RESULTS

### Chromosome-level assembly and annotation of GS

Using a combination of Illumina HiSeq X-Ten reads (90.56×), Nanopore long reads (145.38×), and Hi-C sequencing (229.16×) technologies (Supplementary Figs. S1, S2; and Supplementary Table S1), we generated a chromosome-level genome assembly of GS (Supplementary Fig. S3). The assembly was estimated to be 948 Mb, which was close to the genome size estimated by 17-mer analysis (Supplementary Fig. S4 and Supplementary Table S2). In total, 97.60% of contigs were anchored to 25 chromosomes (Supplementary Fig. S3) with a contig N50 of 2 Mb and scaffold N50 of 37 Mb (Supplementary Table S3). Overall, 52.49% of the genome was identified as repeat elements (Supplementary Table S4). Based on the high-quality genome assemblies, we identified a total of 24194 protein-coding genes in GS (Supplementary Table S5). These characteristics of gene structure, including gene length, exon number/length, coding sequence length, and intron number/length, were compared with those of five other fish species (Supplementary Fig. S5). Using BUSCO v3.0.2 [60], we estimated the coverage of 4584 highly conserved single-copy Actinopterygii genes to be 90% in the assembly (Supplementary Table S6). Ultimately, we generated a high-quality reference genome of GS for subsequent population genomic analysis.

### Genetic diversity and divergence of the two populations

To further investigate the genetic diversity and divergence of these two fish populations, we conducted whole genome resequencing of 46 *Gymnocypris* species, including 23 GS and 23 GE individuals. The mean sequencing coverage was approximately 19.14× per individual (range: 15.75–24.08×, Supplementary Table S7). Using the GATK method [61], we identified approximately 12.33 million single-nucleotide polymorphisms (SNPs) (Supplementary Fig. S6). The GS lineage showed slightly higher genetic diversity than the GE lineage (nucleotide diversity [π]: 3.14 × 10^−3^ *vs.* 2.87 × 10^−3^; Tajima's *D*: 1.097 *vs.* 1.056; linkage disequilibrium [LD] mean *r*^2^: 0.079 *vs.* 0.086) (Supplementary Fig. S7 and Supplementary Table S8). The genome-wide mean population fixation statistics (*F*st) and absolute divergence (*D*_xy_) were 0.03 (95% CI: 0.0297–0.0303) and 0.26 (95% CI: 0.2634–0.2639), respectively, which indicated that these two populations were only weakly structured. The species pairs displayed typical *F*_st_ distributions, with a single large peak centred close to the median score and a tail that represented relatively few regions with heightened divergence (Supplementary Fig. S8).

**Figure 2.**
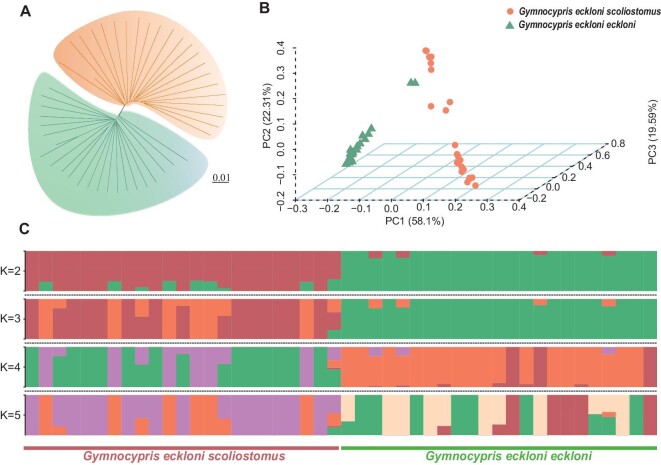
Phylogeny and population structure analysis of *Gymnocypris* species. (A) Neighbour-joining tree based on genome-wide single-nucleotide polymorphisms. Orange branches indicate *Gymnocypris eckloni scoliostomus* and green branches indicate *G. eckloni eckloni*. The scale bar represents the genetic distance between individuals. (B) Principal component analysis of 46 *Gymnocypris* species using whole-genome SNP data. (C) Genetic structure of *G. eckloni scoliostomus* and *G. eckloni eckloni* lineages using the Admixture program. Each accession is represented by a bar, and the length of each coloured segment in the bar represents the proportion contributed by that ancestral population.

GS and GE were identified as two distinct *Gymnocypris* species population clusters based on analysis of all SNPs using the neighbour-joining method (Fig. 2A), principal component analysis (Fig. 2B), and the maximum likelihood method in Admixture v1.3 [62] (Fig. 2C and Supplementary Table S9).

**Figure 3.**
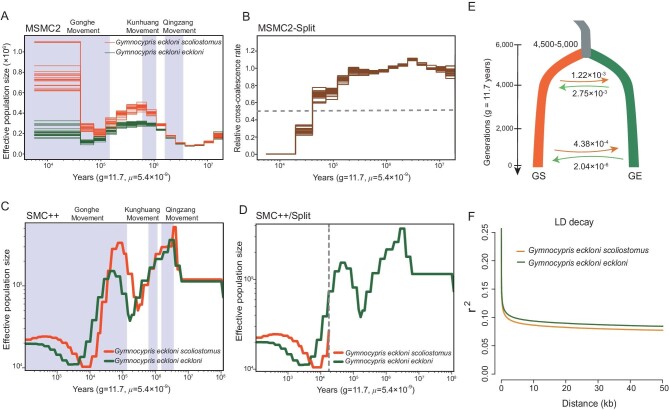
Inferred demographic history of the *Gymnocypris eckloni scoliostomus* (GS) and *G. eckloni eckloni* (GE) lineages. (A) MSMC2-derived demographic history of the GS and GE lineages from 10^4^ to 10^7^ years ago. Each line represents a run of four haplotypes from two individual accessions. (B) MSMC2 split analysis of the GS and GE lineages based on relative cross-coalescence rate. (C) Demographic history of GS (*n* = 23) and GE (*n* = 23) from 10^2^ to 10^8^ years ago based on SMC++ analysis. (D) SMC++ split analysis of the GS and GE lineages. (E) Inferred demographic gene flow of the GS and GE lineages using Fastsimcoal2. Migration rates correspond to 95% CIs obtained from this model. Estimates of gene flow between lineages were provided as migration fraction per generation. (F) Decay patterns of linkage disequilibrium (LD) in GS and GE. Generation time (g) = 11.7 years; neutral mutation rate per generation (μ) = 5.4 × 10^−9^. The time ranges of the three rounds of intense uplift (Qingzang, Kunhuang, and Gonghe movements) are highlighted in light blue on pictures A and C.

The evolutionary divergence of the two lineages was investigated using the multiple sequentially Markovian coalescent (MSMC2) model [63] and SMC++ v1.11 [64]. The GS population showed a higher *N*e than the GE population, and the two subspecies underwent two rounds of population decline during or following three intense uplift phases, including the Qingzang, Kunhuang, and Gonghe movements in the third tectonic uplift of the Qinghai–Tibet Plateau (Fig. 3A, and C). The split analyses showed that the divergence time of the two species was approximately 57 Kya (Fig. 3B) or 20 Kya (Fig. 3D).

### Gene flow

Previous evidence showed that gene flow commonly occurs between recently diverged species despite the existence of barriers to gene flow in their genomes [65]. We used Fastsimcoal v2.6 [66] to simulate and compare five different hypothetical models (Supplementary Fig. S9). Using the Akaike information criterion (AIC) and maximum likelihood algorithm, our dataset supported a scenario of different gene flow matrices in which there was a large amount of gene flow in the early stage of interspecific differentiation, but gradually decreased with the progress of speciation (Fig. 3E; Supplementary Figs. S10, and S11; and Supplementary Table S10). In the early stage of interspecific differentiation, gene flow was estimated to be 1.22 × 10^−3^ per generation (95% CI: 0.0008–0.0011) from the GS lineage to the GE lineage, and 2.75 × 10^−3^ per generation (95% CI: 0.0021–0.0026) from the GE lineage to the GS lineage. In the late stage of speciation, however, gene flow was approximately 4.38 × 10^−4^ per generation (95% CI: 0.0003–0.0004) from the GS lineage to the GE lineage and 2.04 × 10^−6^ per generation (95% CI: 2.05 × 10^−6^–5.54 × 10^−6^) from the GE lineage to the GS lineage (Fig. 3E).

### The pure sympatric speciation model predicts small islands

To determine the characteristics of genomic islands formed in pure sympatric speciation, we carried out computer simulations based on the recurrent selection and backcross (RSB) model [67]. The RSB method, which is based on recurrent selection, and backcross and intercross schemes, was initially proposed for identifying genes in quantitative trait loci. This is accomplished by continuously selecting for breed A traits while backcrossing to breed B. This strategy was also adapted to the speciation-with-gene-flow model by adjusting parameter values (strength of selection, migration rate, recombination rate, and relative fitness of a sequence; see Materials and Methods).

Introgression was simulated in 10 Mb diploid genomes under the conditions of strong selection (s = −0.5) and high migration rate (m = 0.1 for each generation). The simulation results for 1000, 5000 and 10000 generations revealed different degrees of negative selection (Supplementary Fig. S12). More than 99% of the 10 Mb sequence was negatively selected after 10000 generations (Supplementary Fig. S12). The simulation results revealed that, except for the middle region, the sequences on both sides were eventually replaced by more frequent bidirectional migrations in diverging sympatric populations under strong selection. These results indicated that large genomic islands (≥100 kb) between sympatric species are rare, whereas small genomic islands (<100 kb) are widespread. Moreover, the LD decay distance of both species was approximately 10 kb (Fig. 3F), which was consistent with the results of the above model.

### Genomic islands between species

**Figure 4.**
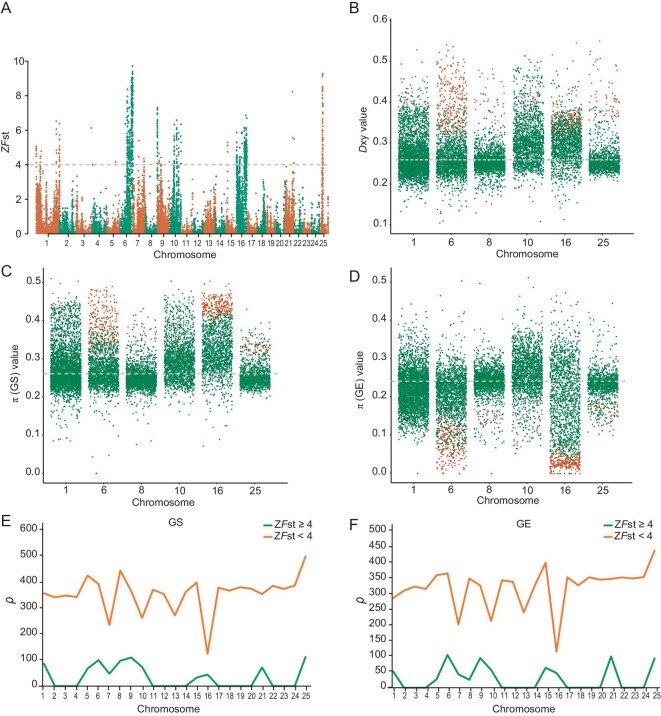
Patterns of genome-wide divergence between *Gymnocypris eckloni scoliostomus* (GS) and *G. eckloni eckloni* (GE) lineages. (A) Genome-wide Z*F*_st_ scores measured in 20-kb non-overlapping windows for GS and GE. Dots above Z*F*_st_ ≥4 were identified as genomic islands. (B) Absolute divergence (*D*_xy_) in genomic islands (red dots) and the rest of the genome (green dots). Compared with the rest of the genome, the genomic islands in these two lineages showed significantly elevated *D*_xy_ (Mann–Whitney U *P* < 2.2 × 10^−16^). (C and D) Nucleotide diversity (π) results in genomic islands (red dots) and the rest of the genome (green dots) in GS (C) and GE (D). The results indicated that π at genomic islands in GS had significantly increased, whereas π at genomic islands in GE had significantly decreased compared to the rest of the genome (Mann–Whitney U *P* < 2.2 × 10^−16^). (E and F) The population-scaled recombination rate (*ρ*) in genomic islands (orange line) and the rest of the genome (green line). The *ρ* for both lineages in genomic islands was considerably lower than that in the rest of the genome (Mann–Whitney U *P* < 2.2 × 10^−16^).

Genomic islands between species pairs were detected by relative divergence (Z*F*_st_) values. In total, 744 (merged into 107 nonoverlapping windows) genomic islands were identified between the two species; 54 of these 107 genomic islands were ≥100 kb and accounted for 89.4% of total genomic island length (Table 1). The most significant genomic islands were on chromosomes 1, 6, 8, 10, 16, and 25, regardless of the calculated Z*F*_st_ in 10- or 20-kb nonoverlapping windows (Fig. 4A and Supplementary Figs. S13, and S14). The two lineages showed significantly elevated *D*_xy_ and reduced population-scaled recombination rate (*ρ*) in these genomic islands compared with the rest of the genome (Mann–Whitney U *P* < 2.2 × 10^−16^) (Fig. 4B, E, and F; Supplementary Figs. S15A, and 15B; and Supplementary Table S11). Intriguingly, we found strongly increased π in the GS lineage and strongly reduced π in the GE lineage in these genomic islands compared with the rest of the genome (Mann–Whitney U *P* < 2.2 × 10^−16^) (Fig. 4C, and D; Supplementary Fig. S15C–S15F; and Supplementary Table S11).

**Table 1.** Number of genomic islands with various lengths from three sets of relative divergence values (Z*F*_st_ ≥4, Z*F*_st_ ≥3.5, Z*F*_st_ ≥3).

Length of genomic islands (kb)	Numbers of genomic islands (Z*F*_st_ ≥4)	Numbers of genomic islands (Z*F*_st_ ≥3.5)	Numbers of genomic islands (Z*F*_st_ ≥3)
20–80	53	72	101
100–200	28	27	26
220–620	19	21	19
640–1000	4	5	1
1160–2020	3	3	8
Total	107	128	155
Number of genomic islands (≥100 kb)	54 (50%)	56 (44%)	54 (35%)
Total length of genomic islands (≥100 kb)	17.91 Mb (89.4%)	19.67 Mb (87.5%)	23.79 Mb (85.9%)
Number of genomic islands (≥220 kb)	26 (24.2%)	29 (22.66%)	28 (18.1%)
Total length of genomic islands (≥220 kb)	13.79 Mb (68.89%)	15.86 Mb (70.58%)	20.25 (73.1%)

In total, we identified 226 genes in these genomic islands. Gene Ontology (GO) analysis showed that these genes were significantly enriched for olfactory receptor activity, voltage-gated potassium channel related, G-protein coupled receptor signalling pathway, response to stimulus, cation channel complex, signal transducer activity, GTPase activity, and protein phosphorylation and dephosphorylation process (Supplementary Tables S12, and S13). Strikingly, these genes were significantly concentrated in olfactory transduction and its related signalling pathway (Supplementary Table S14). In addition, we found significant selection signals in these genomic islands (see Selection signals in GS and GE lineages), which indicated that these islands are potential selection regions.

### The micro-parapatric speciation model predicts large genomic islands

The RSB method [67] was also used to determine the characteristics of genomic islands formed in micro-parapatric speciation. Introgression was simulated in 10 Mb diploid genomes under the conditions of strong selection (s = −0.5) and low migration rate (m = 0.01 for each generation). The simulation results for 1000, 5000 and 10000 generations revealed different degrees of negative selection (Supplementary Fig. S16). These results suggest that large genomic islands (≥100 kb) occur disproportionately in micro-parapatric speciation (Supplementary Fig. S16).

### Selection signals in GS and GE lineages

**Figure 5.**
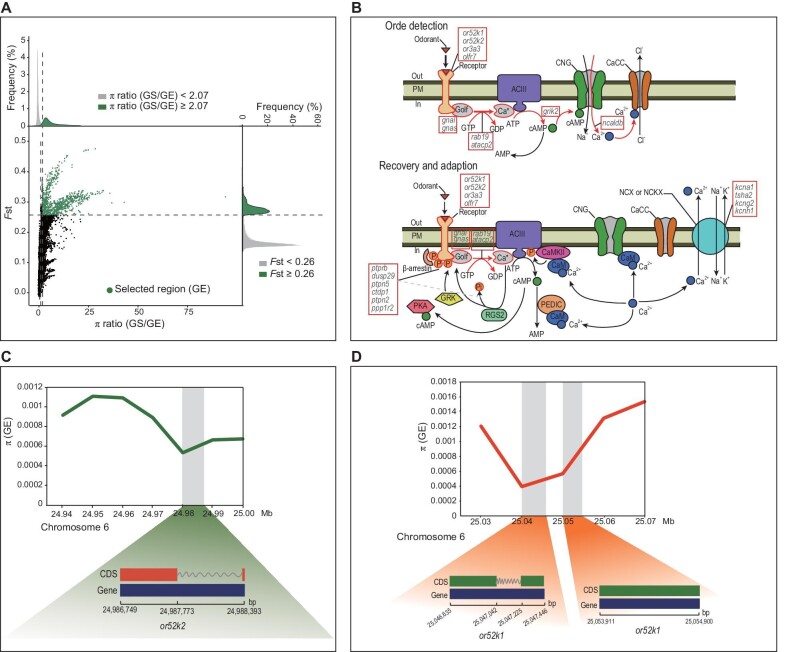
Selected genomic regions of *Gymnocypris eckloni scoliostomus* (GS) and *G. eckloni eckloni* (GE) lineages. (A) Distribution of nucleotide diversity (π) ratio (GS/GE) and *F*_st_ value. The data points located to the right of the right vertical dashed lines are the top 5% of the empirical π ratio distribution (π ratio ≥2.07). Above the horizontal dashed line is the top 1% *F*_st_ distribution (*F*_st_ ≥0.26), which were identified as selection regions for the GE lineage. (B) Olfactory transduction pathway. The olfactory signal processes involved in the selected genes or the genes in the genomic islands are highlighted in the red rectangle. (C and D) π around the selected olfactory receptor genes *or52k2* (green line) and *or52k1* (dark orange line) on chromosome 6. This was measured in 10-kb non-overlapping windows. The grey regions indicate the locations of *or52k2* and *or52k1*.

Despite being closely related, GS and GE are morphologically distinct, especially regarding the shape of the mouth and lower jaw [52]. We used a combination of *F*_st_ and π between the two lineages to explore the selection signals of this differentiation (see Materials and Methods) (Fig. 5A). In total, we identified 187 genes from 1006 putatively selected regions in the GE lineage. The selected genes of the GE lineage were on chromosomes 6, 9, 10, 16 and 25 (Supplementary Fig. S17). Interestingly, no selection signal was found in the GS lineage (Fig. 5A).

Because the selected regions of the top 1% of *F*_st_ values may be too strict, we also identified the windows with the top 5% of *F*_st_ values (*F*_st_ ≥ 0.137) as outlier windows. Surprisingly, only 63 windows including 45 genes were identified in the GS lineage (Supplementary Fig. S18). Compared with GS, these findings indicated that the GE lineage was likely subjected to stronger divergent selection. This was also consistent with the strongly reduced π in the genomic islands of the GE lineage (Fig. 4C, and D; Supplementary Fig. S15C–S15F; and Supplementary Table S11). Importantly, 151 selective sweep genes of the GE lineage (80.2% of selected genes) were located inside identified genomic islands (Supplementary Fig. S19).

GO analysis indicated that the 187 genes were significantly enriched for olfactory receptor activity, protein phosphorylation and dephosphorylation process, voltage-gated potassium channel activity, and ion channel activity (Supplementary Tables S15, and S16). Kyoto Encyclopedia of Genes and Genomes (KEGG) analysis also revealed that these genes were highly enriched in the signalling pathway associated with olfactory transduction (Fig. 5B and Supplementary Table S17). Additionally, π was assessed around the selected olfactory receptor genes *or52k2* and *or52k1* on chromosome 6 (Fig. 5C, and D). The *or52k2* and *or52k1* loci showed strong reduction in π compared with the 40–60 kb genomic region adjacent to this site. This indicates that there is substantial selective advantage of *or52k2* and *or52k1* and that they are not genetic hitchhikers.

In addition, cross population composite likelihood ratio (XP-CLR) test [68] was also used to detect selective sweeps. Using the upper 5% of normalized xpclr values as a cut-off, we identified 1480 genes and 1485 candidate selected genes in the GS and GE lineages, respectively. Enrichment analysis also showed that the selected genes were significantly enriched in the olfactory signalling pathway in GE (Supplementary Tables S18, and S19). However, little enrichment of the olfactory signalling pathway was found in GS. This result is similar to that of the above analysis based on *F*_st_ and π.

Those shared selected genes in GE from the above two approaches were significantly enriched in biological processes, such as response to chemical, response to stimulus, signal transduction, and olfactory receptor activity (Supplementary Table S20). This highlights the selective role of olfaction in GE.

## DISCUSSION

GS and GE are sister species that are sympatrically distributed in Lake Sunmcuo, and they have large differences in morphology (Fig. 1D) and reproductive characteristics. The divergence time between the GS and GE lineages was estimated to be 20–60 Kya (Fig. 3B, and D), which was before Lake Sunmcuo opened into the Yellow River [52]; this indicated that speciation occurred entirely within the lake. It is therefore considered a classic case of sympatric speciation from a biogeographic perspective.

Because of the absence of obvious geographical barriers, bidirectional gene flow was frequent in the early stages of speciation, and the selection intensity had to be sufficiently high to continue differentiation without reversing speciation. The RSB method was used to simulate the pure sympatric model of speciation-with-gene-flow, and the results revealed that small genomic islands (<100 kb) were widespread in sympatric species and large genomic islands were rare (Supplementary Fig. S12). Theoretically, the genomic island size will also be close to the LD decay distance, which was approximately 10 kb (Fig. 3F) for GS and GE. Moreover, recent strong empirical evidence on sympatric mangroves revealed that significant post-speciation gene flow resulted in a large number of introgression blocks averaging only about 3–4 kb in size and non-introgressable genomic islands averaging 1.4 kb in size [69]. Both theoretical and empirical results indicated that large genomic islands are less likely to form in sympatric species (Fig. 1A). Thus, we expected to see numerous different small genomic islands between GS and GE.

In this study, we discovered that genomic islands were concentrated on chromosomes 1, 6, 8, 10, 16 and 25. The genomic islands showed restricted gene flow (high *D*_xy_) and low recombination (low *ρ*) (Fig. 4B, E, and F). In addition, half of the total number of genomic islands were ≥100 kb, and these islands accounted for 89.4% of the total length of all genomic islands (Table 1). This is the opposite of what we expected. The presence of a high proportion of large genomic islands (≥100 kb) indicated that gene flow was largely restricted during speciation. Some scholars have suspected that sympatric speciation may be micro-allopatric speciation in disguise [39,40,70,71]. However, in our study, it is easy to reject micro-allopatric speciation (Fig. 1B) because both genomic islands and non-islands were observed between the two populations, which is different from allopatric speciation, in which geographical barriers completely prevent gene flow. Although the pattern clearly invalidates micro-allopatry, the large genomic islands (≥100 kb) are not compatible with the sympatric model either.

Inversions may also promote the formation of genomic islands [72]. Chromosomal inversions can reduce gene flow through the suppression of recombination, making the accumulation of genetic differences more probable within such inversions [73,74]. Inversion may play a pre-existing role in speciation. If an allele causing significant reproductive isolation is associated with an inversion, the gene flow near that locus will be restricted [75]. However, if the inversions are neutral, their probability of fixation or loss depends purely on population size and migration [76].

Species differentiation is usually related to ecological adaptation, which is the result of natural selection. For example, the apple and hawthorn fly taxa that we previously discussed have resulted, in part, from the sorting of pre-existing ancestral variation, followed by the rapid evolution and substitution of entirely novel host-choice adaptations [73,77]. Thus, this switch onto a novel host was the trigger for speciation [73]. The speciation process of apple and hawthorn flies [78] may not be pure sympatry. In addition, if sympatric speciation's many large genomic islands were the result of inversions, it would require numerous inversions to produce them, yet generating these numerous pre-existing large inversions is extremely difficult.

A lake is typically an exceedingly complex ecosystem with distinct ecological landscapes depending on the lake's vertical water depth. This pattern is consistent with the extensive evidence on parapatric speciation (Fig. 1C): the two subspecies are distributed and carry out essential life activities in different parts of the water column, and the adjacent habitats facilitate divergent selection but also permit gene flow. This micro-parapatry facilitates the formation of large genomic islands (≥100 kb) because habitat preference reduces gene exchange between species, which may favour continued response to selection and thus promote species adaptation. As a result, all evidence showed that GE and GS do not represent a simple case of sympatric speciation but rather micro-parapatric speciation. The RSB simulation also revealed that large genomic islands (≥100 kb) occur disproportionately in micro-parapatric speciation (Supplementary Fig. S16). Recent empirical studies on sympatric speciation [21–38] have not carried out detailed assessments of the relationship between gene flow and genomic islands, even though speciation processes all involved the selection of different habitats and restricted gene flow. Similar to our case, many of those presumed sympatric speciation events may actually be micro-parapatric speciation events because most of those studies examined genomic islands and restricted gene flow [21–38].

Speciation always involves sexual isolation [11,14,79]. Ecological differences may impose barriers to gene flow, and sexual isolation may occur when barriers are strong enough to prevent recent gene flow. Therefore, when assortative mating depends on an ecological character, speciation is not hindered by recombination between mating and ecological loci [11]. Consequently, subpopulations in different habitats may have sufficient intrinsic premating isolation to promote speciation [80]. However, how does RI arise as a correlate of the genetic divergence? The most direct way to answer this question is to identify the differentiated genes. In this study, GE underwent stronger selection than GS, which led to a high level of genomic differentiation (Fig. 4C, and D, and 5A).

Genes related to olfactory receptor activity and olfactory transduction pathways were also found on the genomic islands. Olfactory genes may act as pleiotropic genes that influence habitat and sexual selection. Animals rely strongly on olfaction to locate and identify food sources [81]. Compared with the GS lineage, the GE lineage consumes a wider variety of foods. The GE lineage was discovered to have extensive olfactory signals, and a particularly strong selective effect on three olfactory receptors on chromosome 6 (Fig. 5C, and D). This allowed the GE lineage to occupy more niches in an oligotrophic lake in a short period of time by increasing the variety of food available. However, no significant selected olfactory signal was detected in GS. For fish, olfaction is also crucial in chemosensory communication, which has been related to speciation, particularly in terms of sexual isolation [82–84]. For example, female preference for conspecific males was shown to rely predominantly, if not exclusively, on olfactory cues in Lake Malawi cichlids [85]. Evolution of assortative mating may be the most powerful isolating barrier between ecologically diverging subpopulations [11,86,87].

## CONCLUSION

This study combined theoretical and empirical evidence to provide a new perspective on sympatric speciation. Large genomic islands occurred between the examined subspecies, which revealed reliable evidence that this case of presumed sympatric speciation is actually micro-parapatric speciation.

## MATERIALS AND METHODS

### Sampling and genome sequencing, assembly and variant calling

Fish were collected from Lake Sunmcuo in Jiuzhi, Qinghai, China (101°11′E, 33°38′N) using gill nets and cast nets. GS and GE individuals were identified based on the taxonomic description by Chen and Cao [56]. Genomic DNA was extracted from muscle and liver tissues (frozen in liquid nitrogen) using a DNeasy Blood & Tissue Kit (Qiagen, 69506) in accordance with the manufacturer's protocol. Illumina, Nanopore, and Hi-C libraries were generated using the HiSeq X-Ten platform and GridION X5 DNA sequencers. To assist with genome annotation, RNA was extracted from the heart, liver, kidney, muscle, gill, brain, and gonad tissues (frozen in liquid nitrogen) using an HP Total RNA Kit (Omega Bio-Tek, R6812-00). We annotated the genomes using a combination of *ab initio*, homologous-based gene predictions and RNA-seq [88]. For comparative population genomics analysis, we re-sequenced 46 individuals, including 23 GS and 23 GE individuals, using the HiSeq X-Ten platform (Illumina). The detailed methods of sequencing, assembly, annotation, and SNP calling are provided in the Supplementary Methods.

### Population structure analysis and demographic history estimation

Population structures were investigated using three approaches, including a neighbour-joining phylogenetic tree, a nonparametric principal component analysis, and a full maximum likelihood approach. Species demographic history and divergence time were estimated by MSMC2 [63] and SMC++ [64]. The detailed methods are provided in the Supplementary Methods.

### Gene flow model estimate and genome patterns of genetic divergence

We tested five gene flow models (Supplementary Fig. S8) and used Fastsimcoal v2.6 [66] to infer the dynamic history of gene flow. The AIC was used to identify the best of the five models. The detailed simulation methods are provided in the Supplementary Methods.

Population genomic differentiation was measured by Z*F*_st_ and *D*_xy_. Genomic regions with Z*F*_st_ ≥4 were considered genomic islands. Significance of *D*_xy_, π, and *ρ* were assessed for genomic islands. The detailed estimation methods of Z*F*_st_, *D*_xy_, π, and *ρ* are provided in the Supplementary Methods.

### Population genetic simulations under migration, selection, and recombination

To investigate the influences of migration, selection, and recombination on genomic sequences, we carried out computer simulations based on the RSB model [67]. Pure sympatric speciation and micro-parapatric speciation models were simulated. The difference between the two models is the migration rate in speciation. We set a high level of migration (m = 0.1 per generation) in the pure sympatric speciation model because there was frequent bidirectional gene flow between species. A low level of migration (m = 0.01 per generation) was set in micro-parapatric speciation model. Population size was set to 5000 and the recombination rate (r) was set to 10^−8^ per generation between adjacent base pairs. The length of simulated sequences was 10 Mb (100 kb is the basic unit that cannot be separated by recombination). The recombination probability for a 10 Mb sequence was 0.1. Because population size was 5000, there was an average of 500 individuals with recombination in each generation. The relative fitness of the sequence of a hybrid is 0.5. At the beginning of the simulations, the sequences were in their original state (Supplementary Fig. S12A). After several generations of migration, selection, and recombination, the sequences were shuffled.

### Identification of selected genomic regions

For SNPs, we performed a test for selective sweeps in the GS and GE lineages to identify candidate regions using a cross of π and *F*_st_ approaches; π and *F*_st_ were calculated using VCFtools v1.13 [89] in a 100-kb sliding window with a step size of 10 kb. We identified the window with the top 1% or 5% of *F*_st_ values (*F*_st_ >0.26 and *F*_st_ >0.137, respectively) as the outlier windows. On this basis, π ratio values were used (ratio of π values: π [GS]/π [GE] >2.07, top 5%; π [GS]/π [GE] <0.88, bottom 5%) to identify selected genomic regions in the GE and GS lineages. XP-CLR test [66] was also used to detect selective sweeps using the upper 5% of normalized xpclr values as a cut-off. These protein-coding genes were annotated with GO [90] using the InterPro [91] and eggNOG [92] databases. KEGG annotation [93] used KASS [94] to identify the function of selected genes.

## DATA AVAILABILITY

The raw genome data for *Gymnocypris eckloni scoliostomus* and the genome-resequencing data for *G. eckloni scoliostomus* and *G. eckloni eckloni* have been deposited in the NCBI database under BioProject accession number PRJNA771798. The genome assembly file of *G. eckloni scoliostomus* is available under accession number JAJHNS000000000.

## Supplementary Material

nwac291_Supplemental_File
