# Supplementary material for: Sympatric or micro-allopatric speciation in a glacial lake? Genomic islands support neither
Source: Natl Sci Rev. 2022 Dec 27;9(12):nwac291. doi: 10.1093/nsr/nwac291 (PMC9905650; doi:10.1093/nsr/nwac291)
Supplement: nwac291_Supplemental_File [file nwac291_supplemental_file.docx]

**Supplementary Data for**

**Sympatric or micro-allopatric** **speciation in a glacial lake? Genomic islands support neither**

**Authors:**

Ning Sun^1,6*^, Liandong Yang^1*^, Fei Tian^2^^*^, Honghui Zeng^1*^, Ziwen He^3†^, Kai Zhao^2†^, Cheng Wang^1,6^, Minghui Meng^1^, Chenguang Feng^1,4^, Chengchi Fang^1^, Wenqi Lv^1,6^, Jing Bo^5,6^, Yongtao Tang^2^, Xiaoni Gan^1^, Zuogang Peng^7^, Yiyu Chen^9†^, Shunping He^1,5,8†^

**Affiliations:**

^1^State Key Laboratory of Freshwater Ecology and Biotechnology, Institute of Hydrobiology, Chinese Academy of Sciences, Wuhan 430072, China.

^2^Key Laboratory of Adaptation and Evolution of Plateau Biota, Northwest Institute of Plateau Biology, Chinese Academy of sciences, Xining 810008, China.

^3^State Key Laboratory of Biocontrol, Guangdong Key Lab of Plant Resources, School of Life Sciences, Sun Yat-sen University, Guangzhou, Guangdong 510275, China.

^4^School of Ecology and Environment, Northwestern Polytechnical University, Xi’an 710129, China.

^5^Institute of Deep-Sea Science and Engineering, Chinese Academy of Sciences, Sanya 572000, China.

^6^University of Chinese Academy of Sciences, Beijing 100049, China.

^7^Key Laboratory of Freshwater Fish Reproduction and Development (Ministry of Education), Southwest University School of Life Sciences, Chongqing 400700, China.

^8^Center for Excellence in Animal Evolution and Genetics, Chinese Academy of Sciences, Kunming 650223, China.

^9^National Natural Science Foundation of China, Beijing 100085, China.

^*^ **These authors contributed equally**

^†^**Corresponding author:** Ziwen He: heziwen@mail.sysu.edu.cn

Kai Zhao: zhaokai@nwipb.cas.cn

Yiyu Chen: chenyy@nsfc.gov.cn

Shunping He: [clad@ihb.ac.cn](mailto:clad@ihb.ac.cn)

# Supplementary Methods

**Genome sequencing and assembly**

Genomic DNA was extracted from muscle tissue (frozen in liquid nitrogen) using the DNeasy Blood & Tissue Kit (Qiagen, 69506) following the manufacturer’s protocol. A 150-bp paired-end library with insert sizes of 300 bp was constructed according to the Illumina protocol and sequenced on the HiSeq X-Ten platform (Illumina). Nanopore libraries were generated according to the manufacturer’s instructions and sequenced on 13 flow-cells using the GridION X5 DNA sequencer (Oxford Nanopore). Hi-C (high-throughput chromosome conformation capture) libraries were constructed and sequenced using a HiSeq X-Ten platform to obtain 150-bp paired-end reads. To assist the genome annotation, RNA was extracted from the heart, liver, kidney, muscle, gill, brain, and gonad using an HP Total RNA Kit (Omega Bio-Tek, R6812-00). These RNA was pooled together to construct a cDNA library, and then sequenced on a Pacific Biosciences RSII sequencer.

We firstly used SOAPec v2.0 [95] to count the kmer frequency with a kmer size of 17. Genome size was then estimated according to the formula: genome size = k-mer number/k-mer depth, where k-mer number is the total number of k-mer from the sequencing data, and k-mer depth is the average depth of 17-mer.

Nanopore high-quality reads were first corrected using the program Canu v1.5 [96] and then assembled with the genome assembler Flye v2.6 [97]. The initial draft assembly was polished three times using Racon v1.2.1 [98] with the raw Nanopore reads, and was further corrected for three rounds using Pilon v1.21 [99] with the Illumina reads. We used HiC-Pro [100] to performed quality control of Hi-C raw data. Finally, the polished contigs were anchored by Hi-C reads using an end-to-end algorithm in Bowtie2 v2.25 [101], and then the assembled contigs were clustered, ordered, and directed onto the 25 pseudochromosomes using LACHESIS v1.0 [102] with default parameters. The completeness of the final assembly was evaluated using BUSCO v3.0.2 [60] with the library “actinopterygii_odb9”.

**Genome annotation**

We annotated the repeat elements in the genome of *Gymnocypris eckloni scoliostomus* (GS) using a combination of *ab initio* and homologous-based gene predictions [88]. Repeat elements of the genome were annotated using RepeatModeler v1.0.5 [103], RepeatMasker v4.0.6 [103] and RepeatProteinMask v1.36 [104]. RepeatModeler was used to run a *de novo* prediction of the repeats, while RepeatMasker and RepeatProteinMask were used to identify repeats by mapping the DNA and protein sequences of repeats from a custom library to the genome. Repeats Finder v4.07 [105] was used to match tandem repeat elements with the parameter: 2 7 7 80 10 50 500 -d -h -ngs.

After the repeat sequences were identified and masked from the genome assembly, we annotated protein-coding genes in the GS genome by combining the results from *ab* *initio* predictions, homologous mapping, and RNA-seq alignment. For *ab* *initio* predictions, Augustus v3.2.1 [106], GeneID v1.4 [107], GlimmerHMM v3.0.4 [108], and SNAP v6.0 [109] were used. For homologous mapping, the protein sequences of another five species (*Danio rerio, Ictalurus punctatus*, *Megalobrama* amblycephala, *Oreochromis niloticus* and Triplophysa siluroides) from ENSEMBL were aligned to the assembly with tBLASTN v2.2.26 [110]. GeneWise v2.4.1 [111] was then used to predict the potential gene structures on all alignments. For RNAseq alignment, reads from Illumina sequencing were aligned to the genome sequences using BLAT v34 [112]. PASA [113] was used to link the spliced alignments. Finally, EVidenceModeler v1.1.1 [114] was performed with default parameters to integrate the three gene sets. Protein sequences of each gene were used for gene functional annotations by mapping against known databases, including the Protein Sequence Database (Nr), Gene Ontology (GO), InterProScan, and Kyoto Encyclopedia of Genes and Genomes (KEGG) with BLASTP v2.2.26 [112].

**Population genome sequencing and variant calling**

For each sample, genomic DNA was extracted from muscle tissue (frozen in liquid nitrogen) by a Qiagen DNeasy Blood & Tissue Kit. All samples were sequenced on the HiSeq X-Ten platform (Illumina). Sequencing libraries were constructed according to the manufacturer’s introductions (Illumina). To filter low-quality reads resulting from base-calling duplicates and adapter contamination, we carried quality control and removed reads according to the following criteria: (i) reads with adapters (ii) reads with ≥ 10% unidentified nucleotides (N); (iii) reads with > 10 nt aligned to the adapter sequence, allowing ≤ 10% mismatches; (iv) reads with > 50% bases having Phred quality < 10. A total of 753 Gb high-quality paired-end reads were retained for downstream data analysis. The qualified paired-end reads were mapped to the GS genome using BWA-MEM v0.7.12 [115]. The bam file of each individual was sorted and indexed by SAMtools v1.9 [116]. The PCR duplicates were masked and removed using in-house Perl scripts. We used GATK v4.1 [61] to call variants using the HaplotypeCaller method based on the HMM likelihood function to create an individual specific gVCF file. All individual gVCF files were combined to generate one gVCF file using CombineGVCFs method, which was then converted to VCF file using GenotypeGVCFs method. We filtered single nucleotide polymorphisms (SNPs) using GATK v4.1 [61] and VCFtools v1.13 [89] according the following criteria: (i) mean sequencing depth (for all individuals) > 5, minor allele frequency > 0.05, missing alleles of per site < 0.1; (ii) QUAL < 30.0, QD < 2.0, FS > 60.0, MQ < 40.0, MQ < 40.0; (iii) SNPs deviating from Hardy-Weinberg Equilibrium (HWE) (P < 0.01) were excluded.

**Estimation of mutation rates**

In addition to the GS, the genome data of another seven fish species (*Danio rerio, Cyprinus carpio*, *Sinocyclocheilus grahami*, *Ctenopharyngodon* idella, *Hypophthalmichthys molitrix*, *Megalobrama amblycephala* and *Schizothorax oconnori*) were collected from ENSEMBL and NCBI to estimate the mutation rates. First, OrthoFinder v2.3.4 [117] with default parameters and RBH method were used to cluster the homologous genes. In total, 288 one-to-one orthologous genes were identified and aligned using MAFFT v7 [118], and the protein-coding sequences were also aligned by the program MAFFT [118]. Second, the orthologous genes alignments were used to construct the phylogenetic trees by IQ-TREE v2.1.2 [119]. Finally, we used MCMCtree implemented in PAML [120] to estimate the average mutation rate per site per generation with the calibration time taken from http://www.timetree.org/. Four softbound calibration time points of fossil dating were applied: *Cyprinus carpio*/*Sinocyclocheilus grahami* (21–46 Mya), *Hypophthalmichthys molitrix*/*Megalobrama amblycephala* (6.8–20 Mya), *Cyprinus carpio*/*Danio rerio* (68–102 Mya) and *Hypophthalmichthys molitrix*/*Ctenopharyngodon* idella (9.3–21.4 Mya).

**Population structure analysis**

Population structures were investigated using three strategies. The first strategy is the neighbor-joining method [121]. A neighbor-joining phylogenetic tree was reconstructed using the nucleotide p-distance matrix with 1000 bootstraps in TreeBest v.1.92. The second strategy is the nonparametric principal component analysis (PCA). The variant calling format was converted to binary ped format using VCFtools v1.13 [89] and PLINK v1.07 [122]. A PCA plot was conducted using the library “ggplot2” in R v4.03. The third strategy is the maximum likelihood algorithm, which is implemented in the Admixture [62] program. This can help to estimate individual ancestry. We set predefined genetic clustering to K = 2–5 to explore potential population division.

**Linkage disequilibrium (LD) analysis**

To estimate the LD patterns of the two populations, we calculated the mean r^2^ values for pairwise markers with a minor allele frequency (MAF) greater than 0.05 using PopLDdecay [123]. Two populations were separated, and SNPs in each population were extracted to perform the analysis. These parameters were set to “-MaxDist 500 -MAF 0.05 -Het 0.88 -Miss 0.2”.

**Genome-wide patterns of genetic diversity and divergence**

The average pairwise nucleotide diversity (π) and Tajima’s *D* statistic of each population were calculated using VCFtools v1.13 [89] with a 100 kb sliding window in 10 kb steps with the parameters “-maf 0.05 -max-missing 0.8 -minDP 4”. Population differentiation was measured using relative (*F*st) and absolute (*D*xy) divergence across the genome in 20 kb nonoverlapping windows. The scripts provided by Simon Martin (https://github.com/simonhmartin/genomics_general) were used for this analysis. The population scaled recombination rate (*ρ* = 4*Ner*) was calculated using FastEPRR [124]. Per-window *F*st values were then standardized to a Z-transformed (Z*F*st) to be able to compare genomic landscapes of divergence among pairs with different divergence times using the following formula: Z*F*st = (*F*st - μ *F*st) / σ *F*st, where *F*st is the *F*st in a window, μ *F*st is an average *F*st over all windows, and σ *F*st is a standard deviation of *F*st values of all windows [125]. Genomic regions with Z*F*st ≥ 4 were considered genomic islands. Genomic regions with Z*F*st ≥ 3.5 or ≥ 3 was also evaluated. Adjacent significant windows were merged. Significance of *D*xy, π and *ρ* of genomic islands were assessed compared with the genomic background (Mann–Whitney U [MWU]). These protein-coding genes were annotated with GO [90] using the InterPro [91] and eggNOG [92] database. KEGG annotation [93] used KASS [94] to identify the function of selected genes.

**Estimation of demographic history**

The multiple sequential coalescent Markovian model (MSMC) method [63] was used to simulate the history of the two genetic groups and infer the historical changes in their effective population sizes and population separations. The input files for MSMC were generated using MSMC Tools (https://github.com/stschiff/msmc-tools). We applied this method to the two lineages, each of which has two deep-coverage (> 20 ×) individuals. For each individual, only sites with uniquely mapped reads and sites with coverage depths equal to or greater than 5 were used in the analyses. All sites were phased using Beagle v5.2 [126]. For effective population size inference, two individuals (4 phased haplotypes) from each population were used. We defined the estimated divergence time between a pair of populations as the first time point at which the cross-coalescence rate was at or above 0.5. Demographic history inference was also employed using SMC++ v1.11 [64]. Two populations of total 46 individuals were used. The fixed per-generation mutation rate at 5.4 × 10^−9^ per base pair and a constant generation time of 11.7 years were employed in this study.

**Gene flow model estimate**

We used Fastsimcoal v2.6 [66] to infer the dynamic history of gene flow between the GS and *Gymnocypris eckloni eckloni* (GE) lineage. We used the python script easySFS.py (https://github.com/isaacovercast/easySFS) to generate a two dimensional-folded site frequency spectrum (SFS). Five gene flow models were compared in this analysis (Supplementary Figure S9). For each replicate of each model, Fastsimcoal will perform 200,000 coalescent simulations (-n) to approximate the expected SFS in each cycle and ran 50 optimization (EMC) cycles (-L) to estimate the parameters. We specified a mutation rate of 5.4 × 10^−9^ per site per generation following the estimates. Here we set the split time between the populations to 6000 generations (g=11.7 years). We ran each model 100 times to select the model with the highest likelihood as the best run. We then assessed the model's best fitting parameter. We assessed the fitness of the different models by comparing residuals between the observed SFS and expected SFS (Supplementary Figure S10). The Akaike information criterion (AIC) was used to identify the best model among the five models. However, if the SNPs are not independent, the AIC method can overestimate the support for the most likely model. We also obtained likelihood distributions for each model with the best parameters by iterating 100 times in order to determine whether it has a good fit to the observed data (Supplementary Figure S11). For the selected best model, we used block-bootstrapping to account for linkage between SNPs. We performed 50 bootstrap replicates to determine our parameter estimate certainty.

# Supplementary Figures


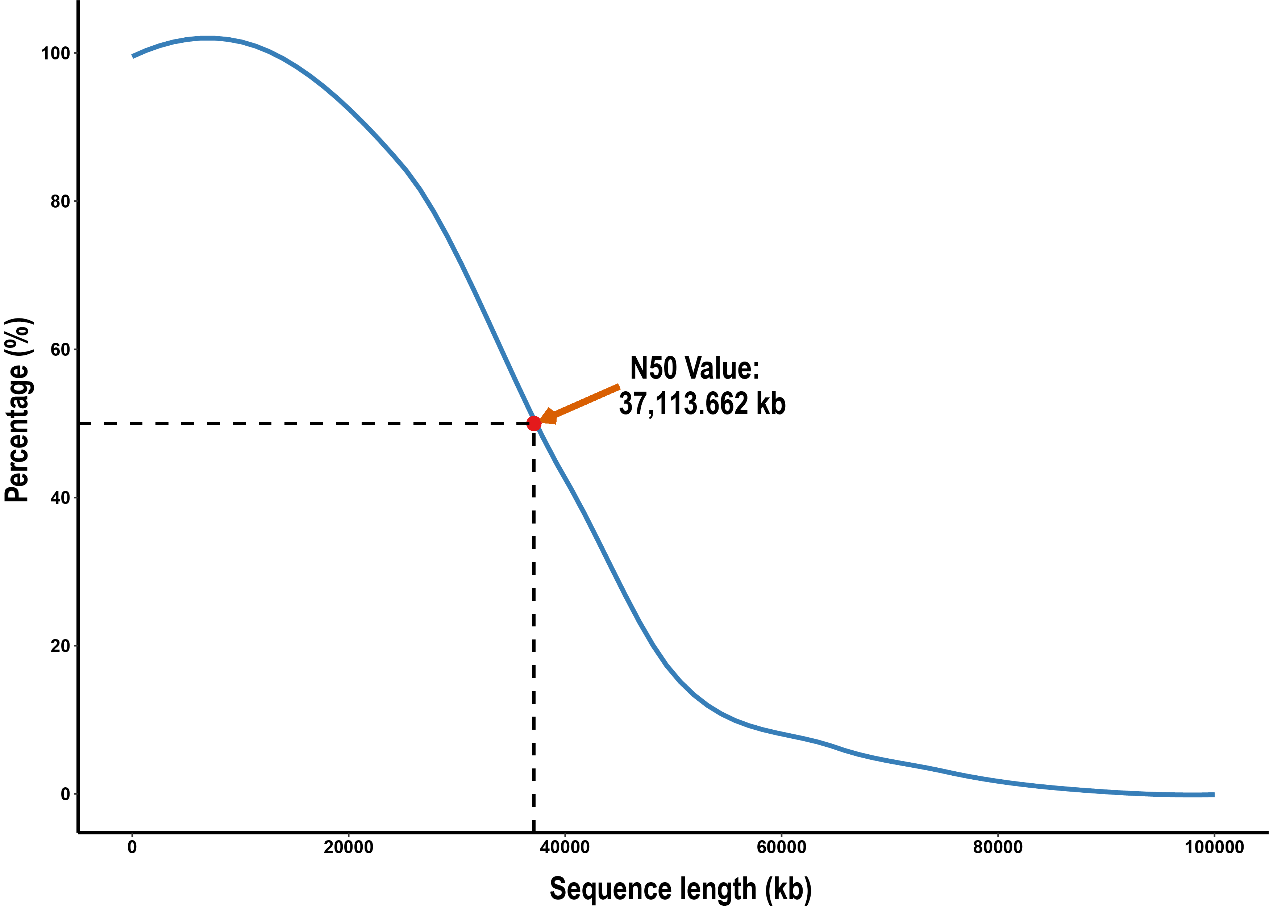


Supplementary Figure S1. Cumulative length distribution of the reads in all sequel runs. The intersection of the two dashed lines indicates the position of the N50, which is 37,113.662 kb of *Gymnocypris eckloni scoliostomus*.


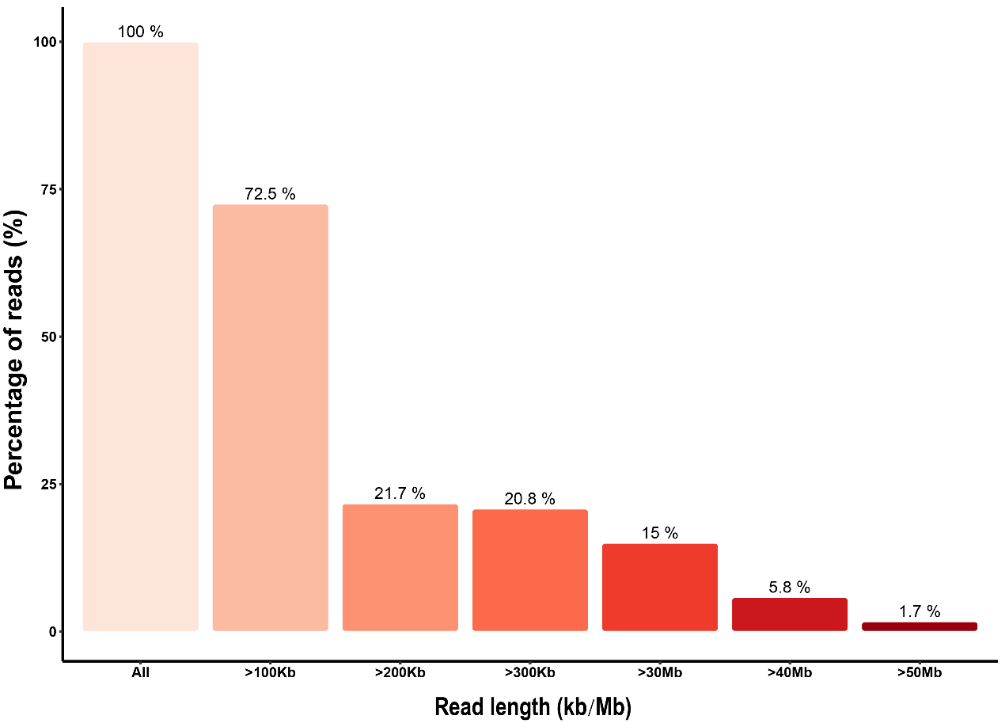


Supplementary Figure S2. Reads length distribution showing percentages of reads with 100 kb, 200 kb, 300 kb, 30 Mb, 40 Mb and 50 Mb. 72.5% of all produced reads ≥ 100 kb for *Gymnocypris eckloni scoliostomus*.


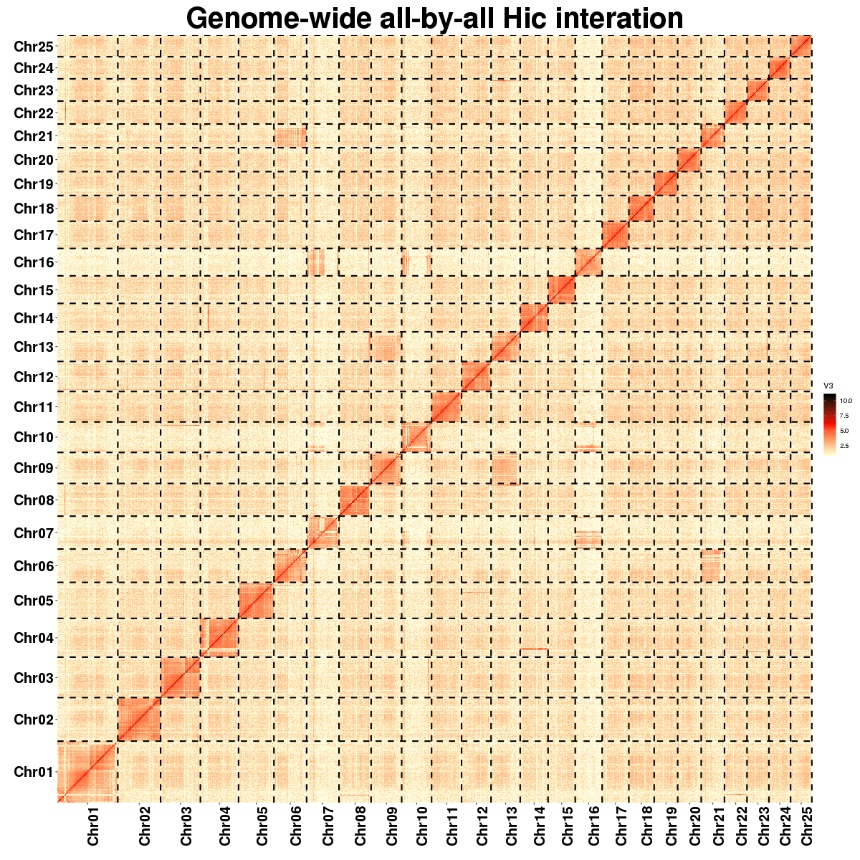


Supplementary Figure S3**.** Chromosome assembly of *Gymnocypris eckloni scoliostomus* using the interaction frequency from the Hi-C data. The color represented the logarithm of the interaction density from high (red) to low (white) in the plot.


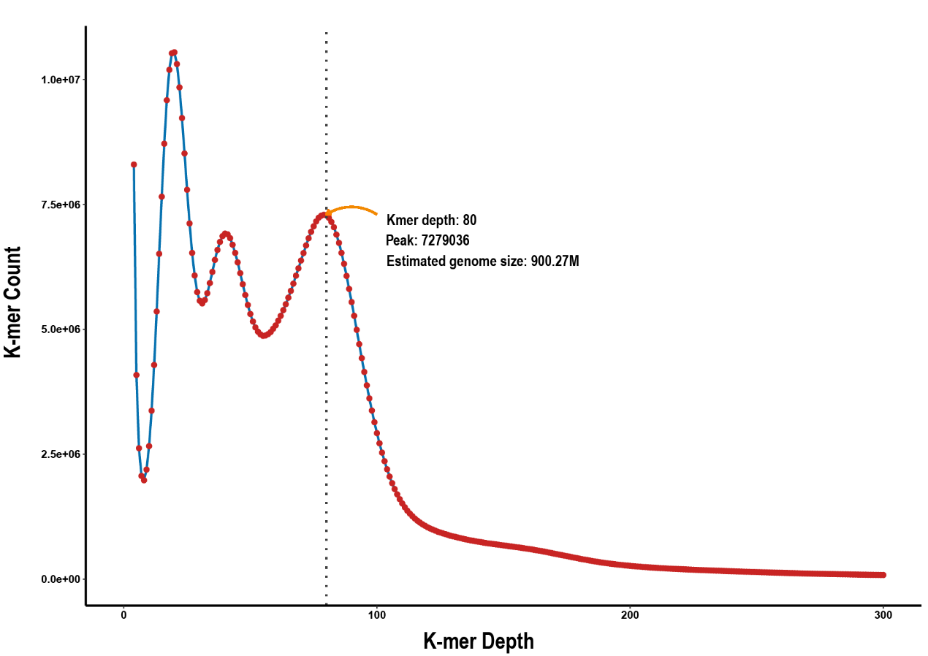


Supplementary Figure S4**.** Results of 17-mer frequency analysis to estimate the *Gymnocypris eckloni scoliostomus* genome size. The x-axis represents depth (X); The y-axis represents the proportion of the frequency at that depth divided by the total frequency of all depths.


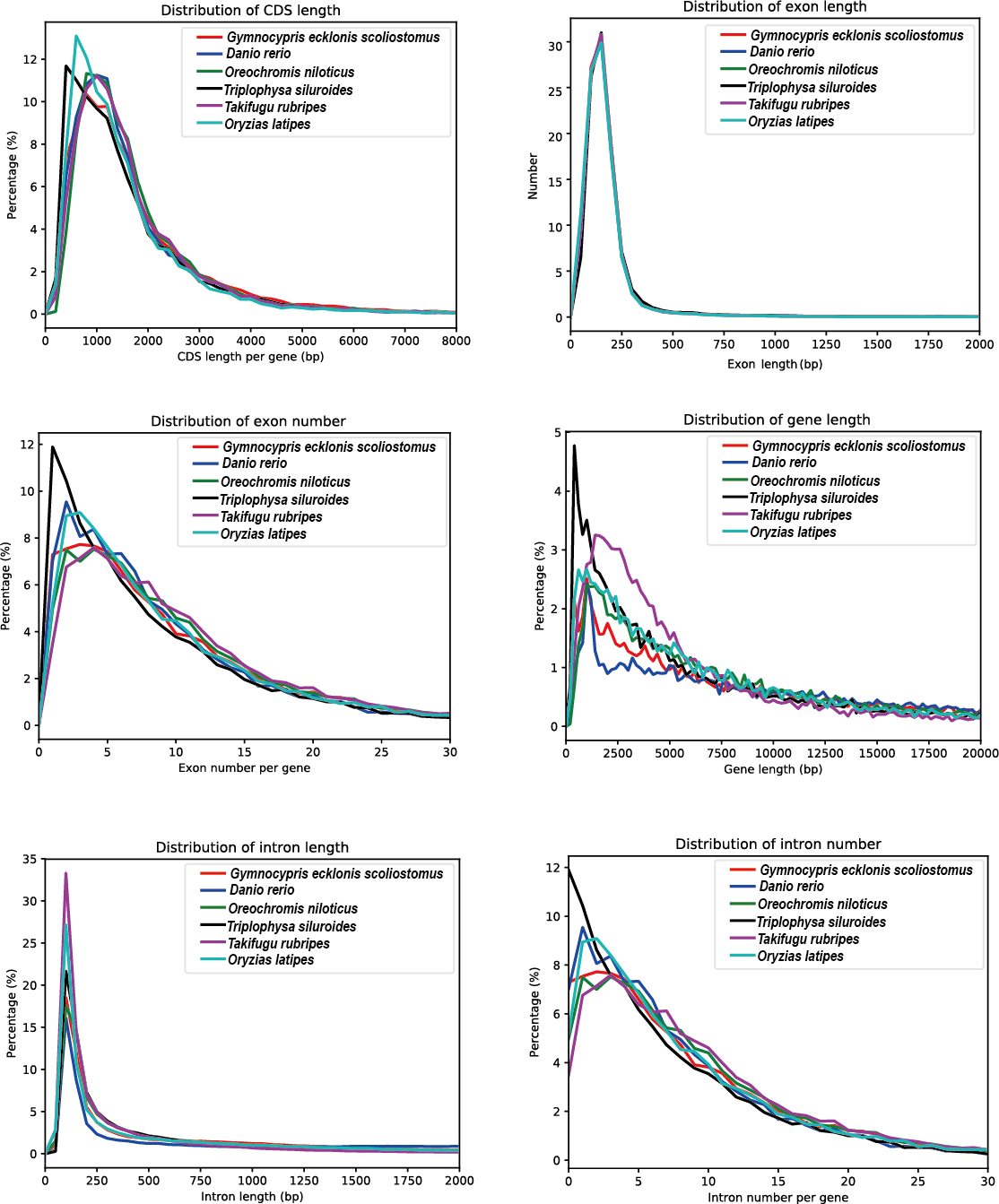


Supplementary Figure S5**.** Comparison of gene structure features of *Gymnocypris eckloni scoliostomus* with other five fish species. The gene length, exon number/length, the coding sequences (CDS) length and intron number/length, were comparable to those of another five fish species.


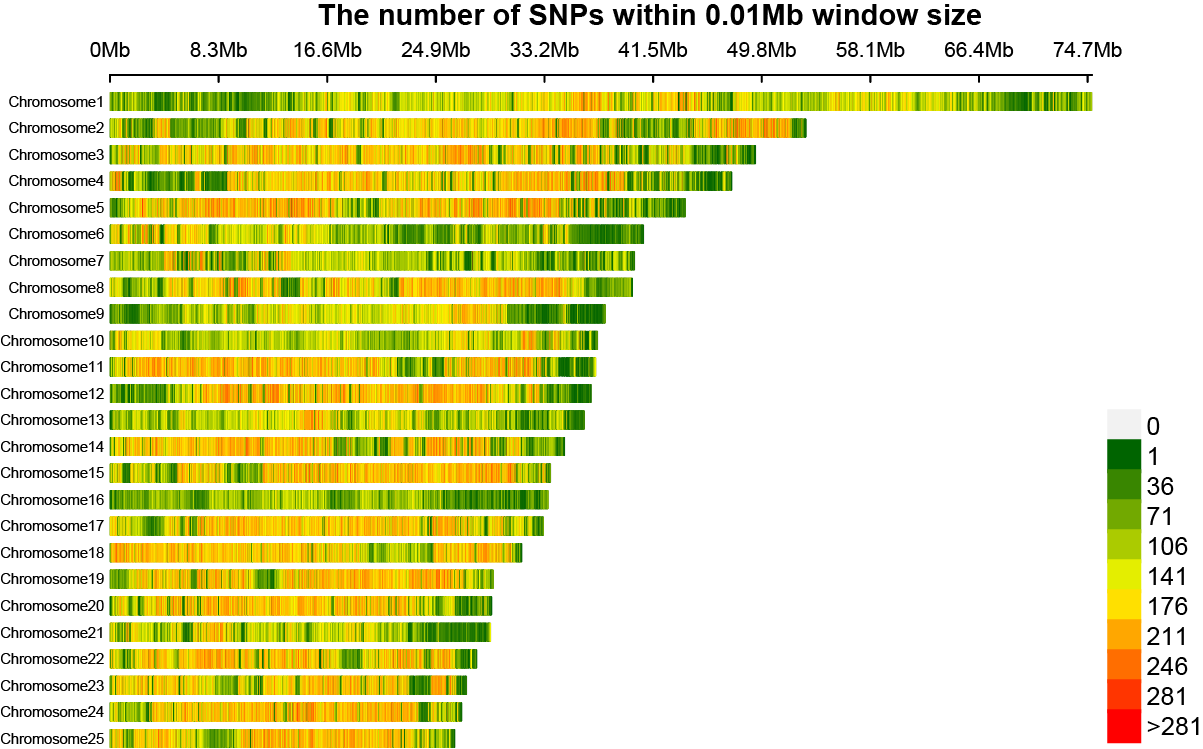


Supplementary Figure S6**.** Panoramas of the SNP density across 25 chromosomes with contiguous 0.01Mb subregions.

**
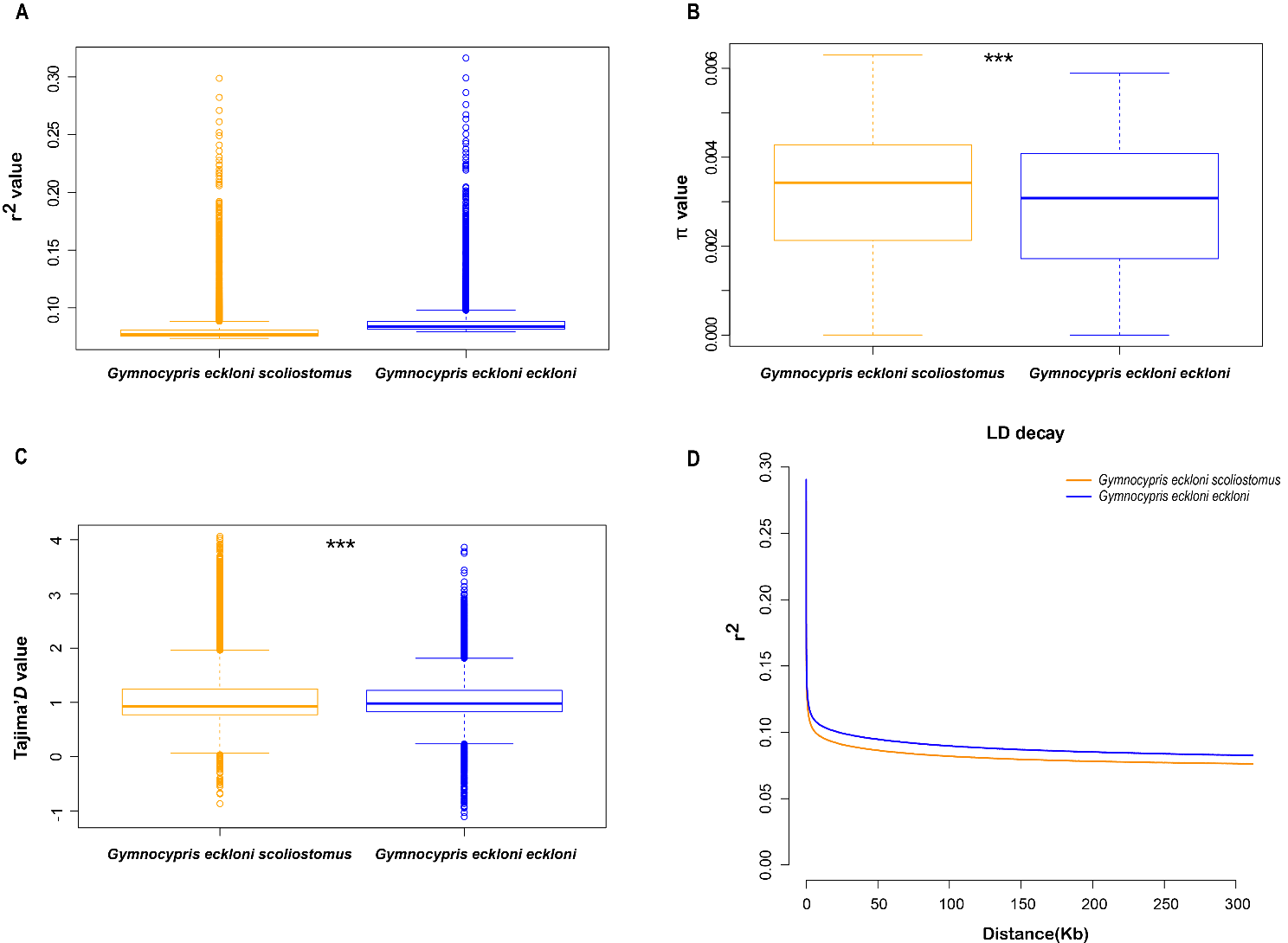
**

Supplementary Figure S7**.** Nucleotide diversity (π), Tajima’s *D*, linked disequilibrium (LD) values (r^2^), and LD decay patterns of the two *Gymnocypris* species. *** indicate Mann–Whitney U P < 6.8×10^-13^.


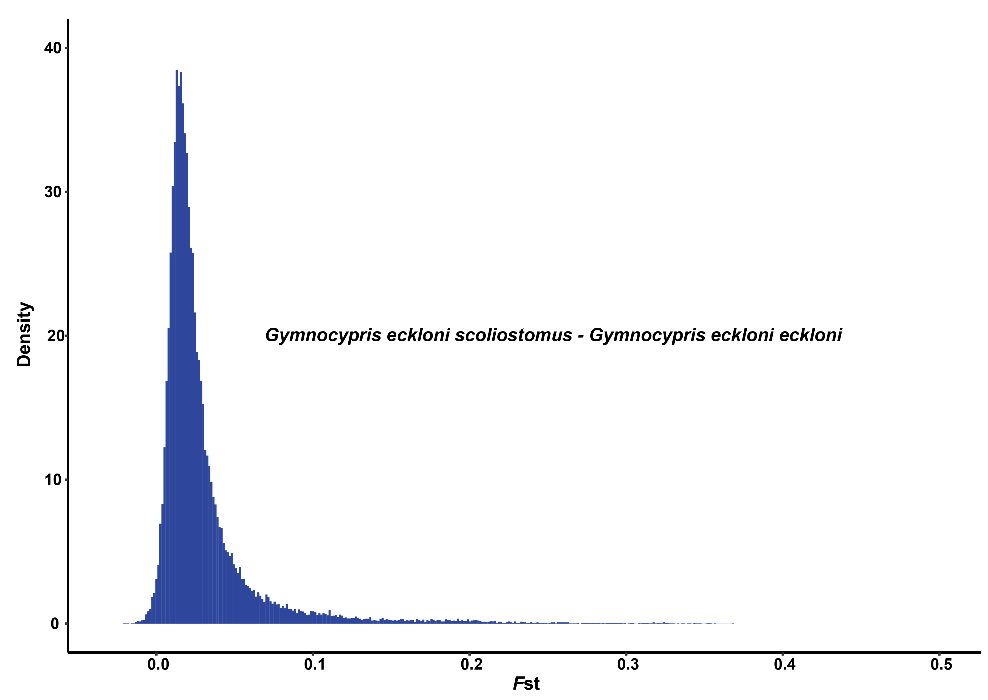


Supplementary Figure S8**.** Histograms showing distributions of *F*st measured in 20 kb windows across the genome for a comparison of *Gymnocypris eckloni scoliostomus* (n = 23) and *G. eckloni eckloni* (n = 23). The species pairs displayed typical *F*st distributions, with a single large peak centred close to the median score and a tail that represented relatively few regions with heightened divergence.


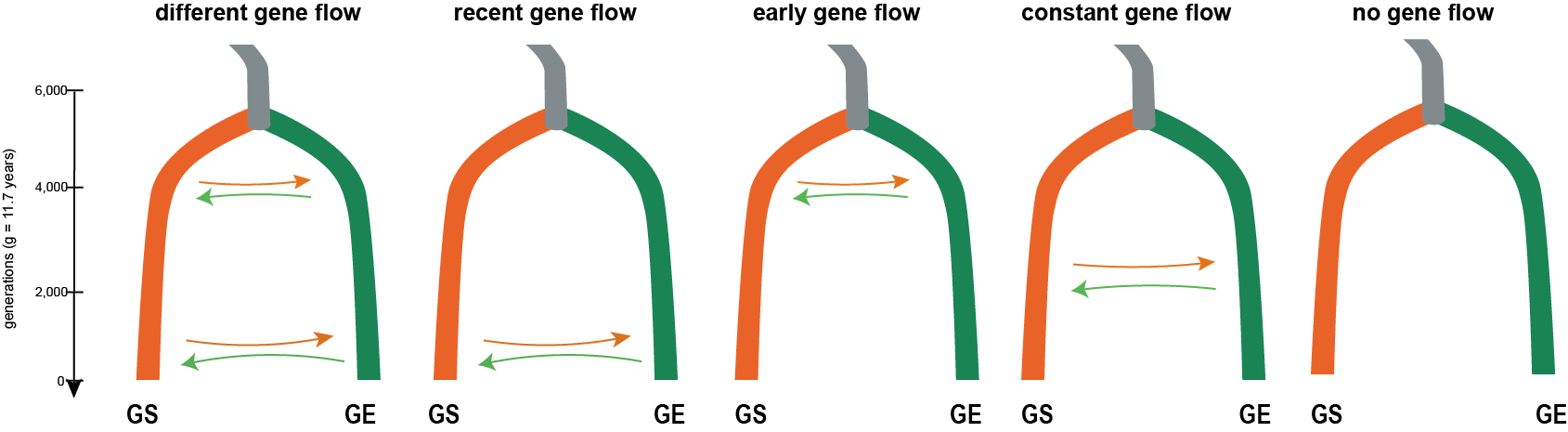


Supplementary Figure S9**.** Detailed schematics of the five demographic models compared in this study. Arrows indicate the direction of gene flow. GS: *Gymnocypris eckloni scoliostomus*; GE: *G. eckloni eckloni*.


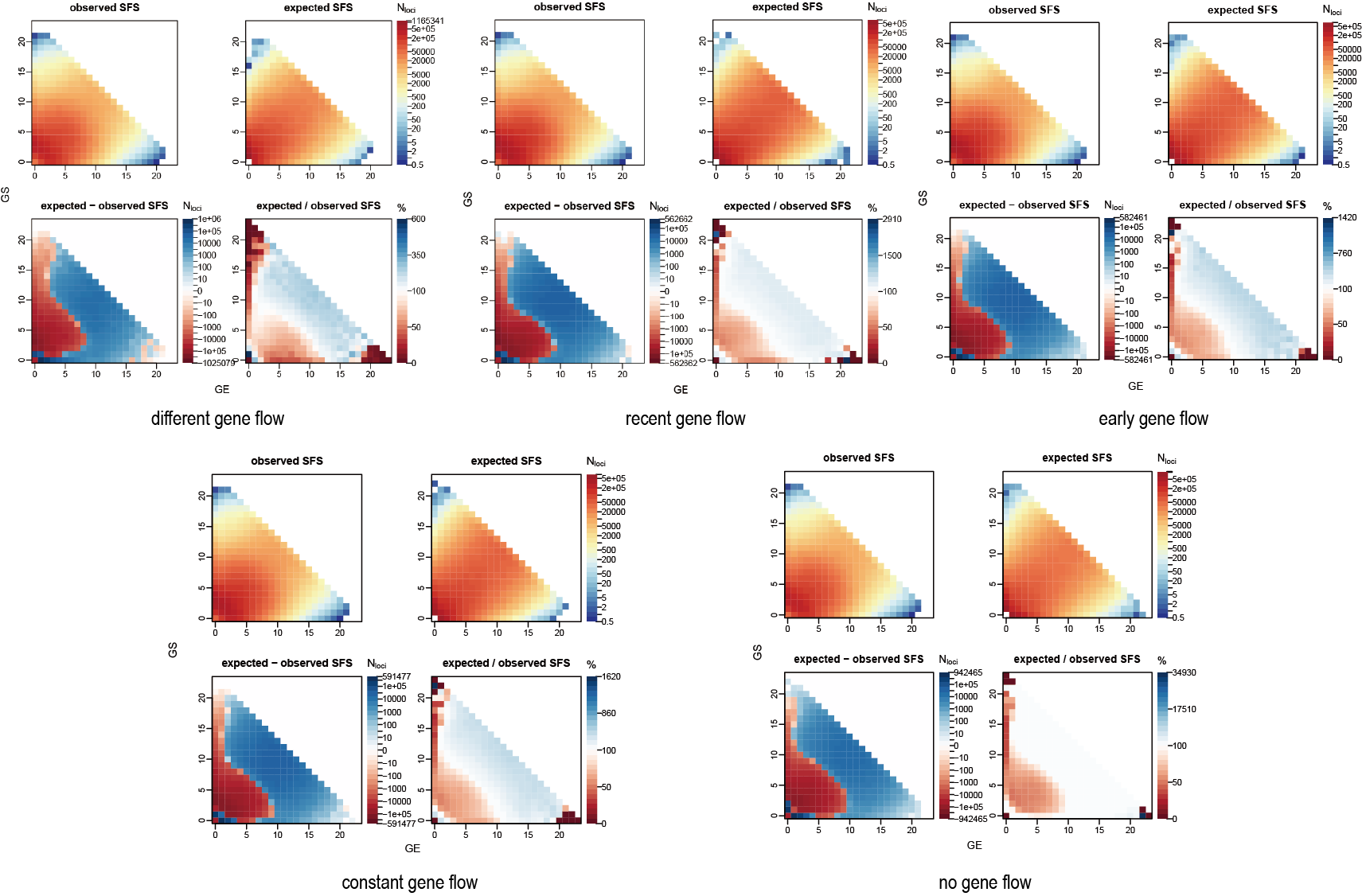


Supplementary Figure S10**.** Pairwise marginal 2-D SFS fit assessment for the five gene flow models from the Fastsimcoal analysis.


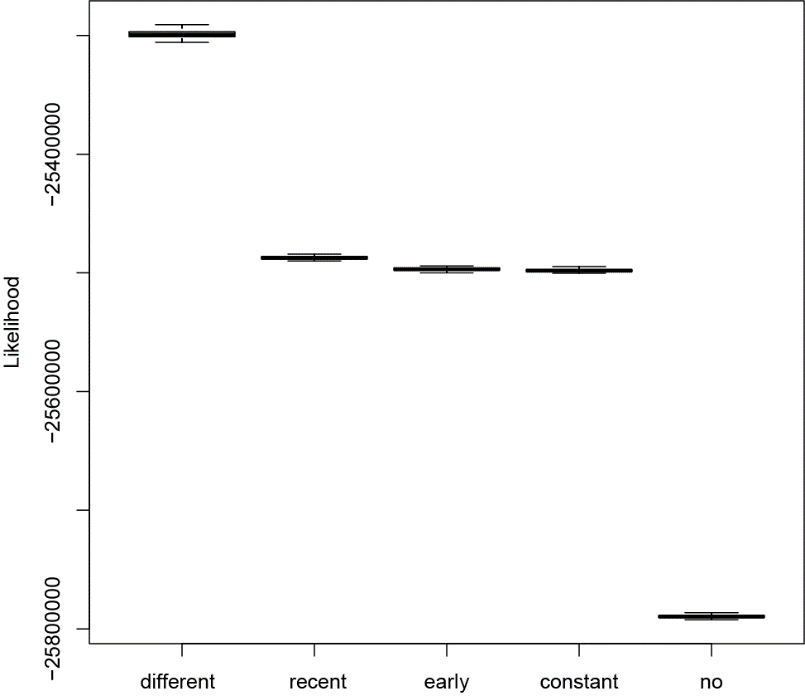


Supplementary Figure S11**.** Likelihood distributions for the five gene flow models with the best parameter values.


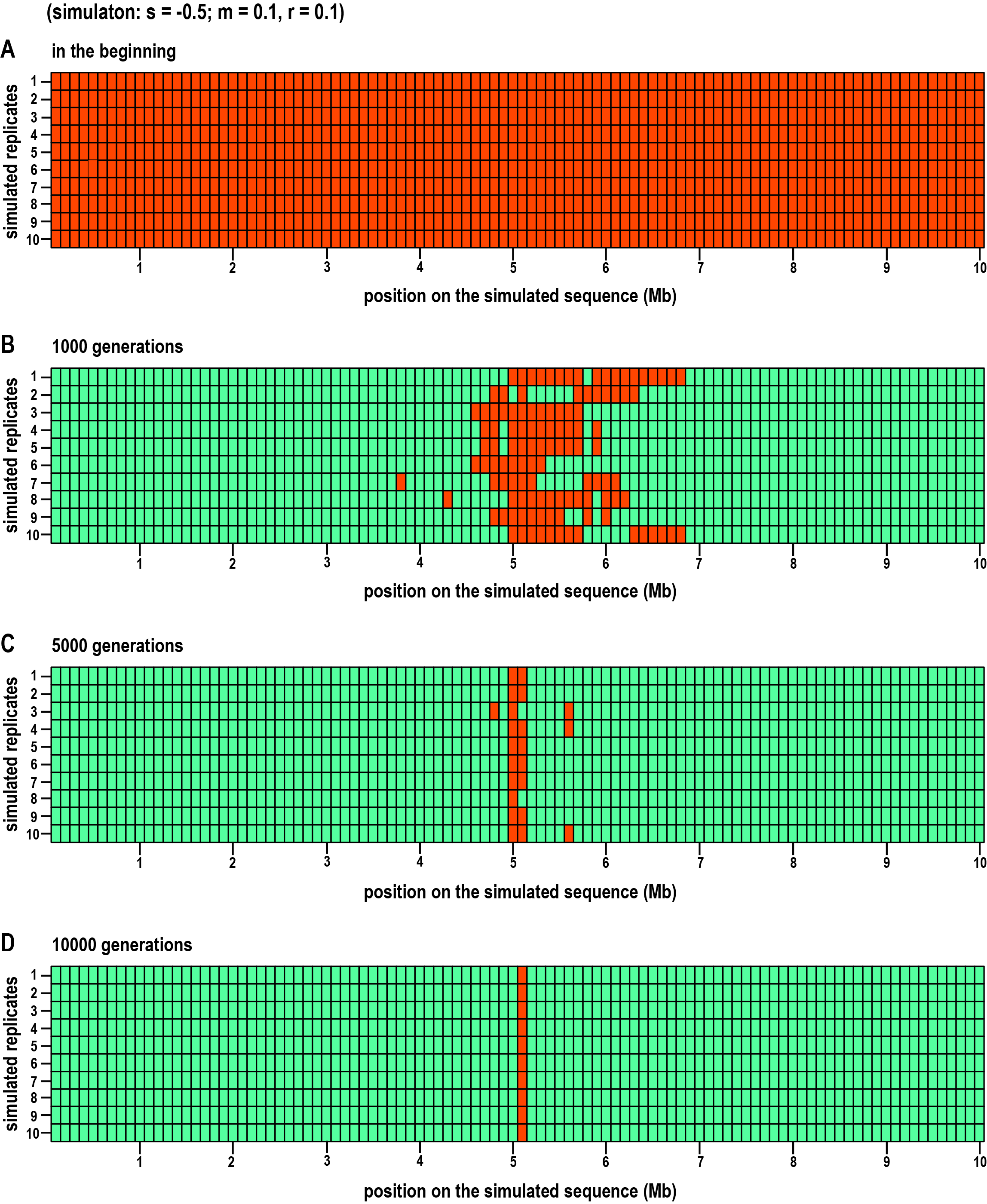


Supplementary Figure S12. Simulated introgressions in diploid 10 Mb genomes. Red and green represent sequences from two different species. The sequences of the two species may differ or not. (A). In the beginning, the sequences were in original sequence states. (B-D) Simulated results of 1,000, 5,000, 10,000, generations under strong selection (s = − 0.5), strong migration rate (m = 0.1), recombination rate (r = 0.1 for per 10 Mb per generation). These results implied that the genome islands size in pure sympatric speciation should be less than 100 kb.


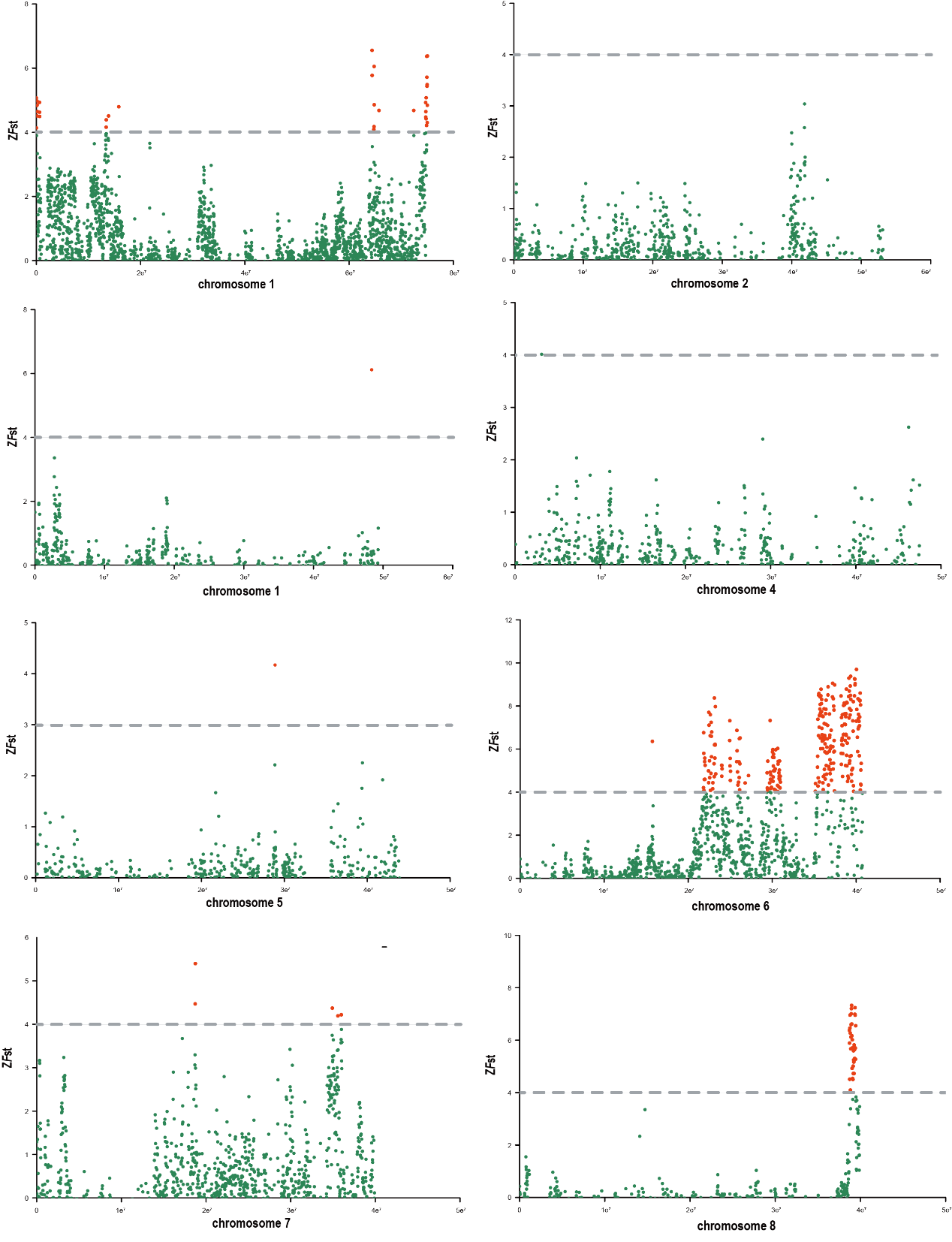


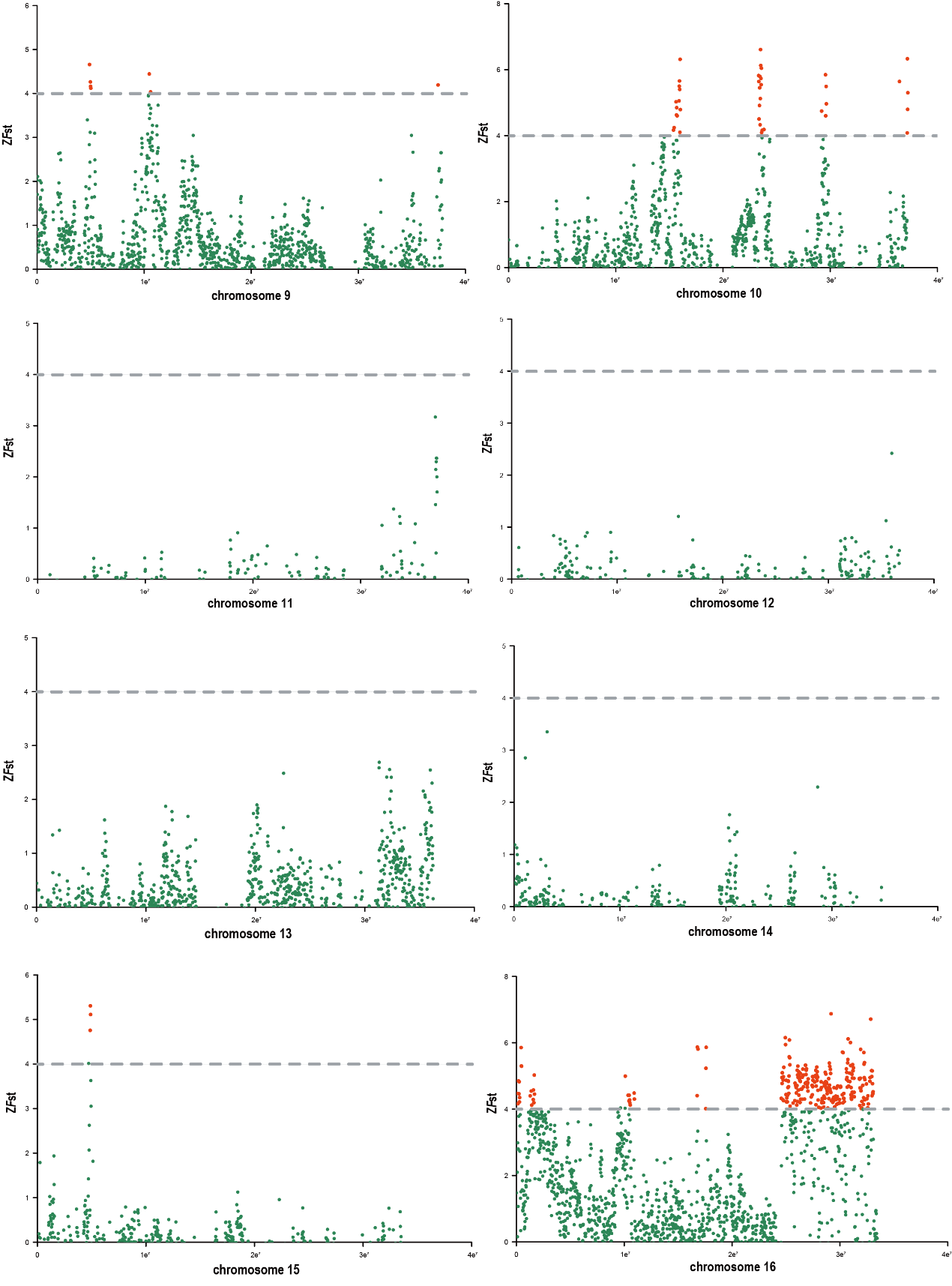


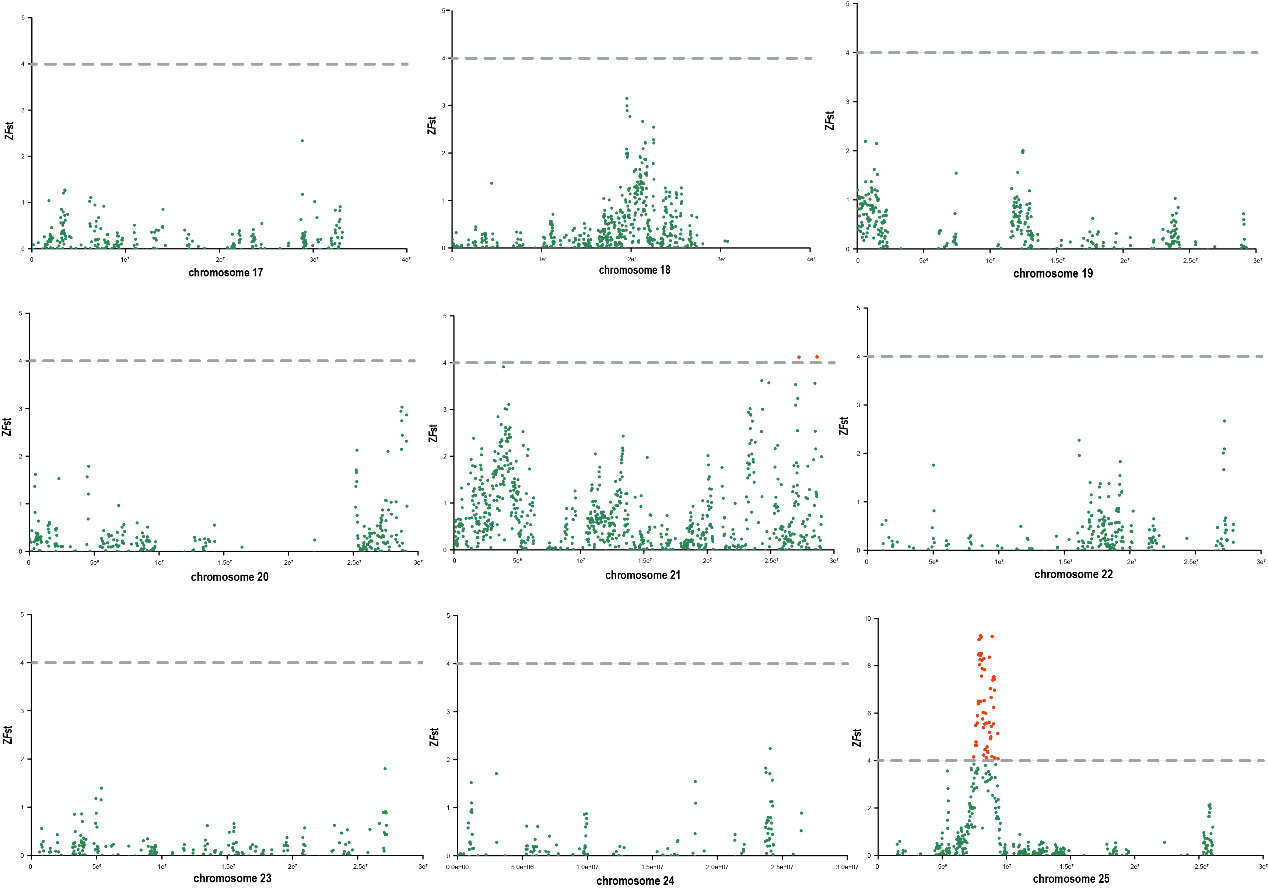


Supplementary Figure S13**.** Z-transformed pairwise genetic divergence (Z*F*st) scores calculated from 10 kb sliding windows across all chromosomes for *Gymnocypris eckloni scoliostomus* (GS) and *G. eckloni eckloni* (GE) comparison. Genomic islands are shown as red dots.


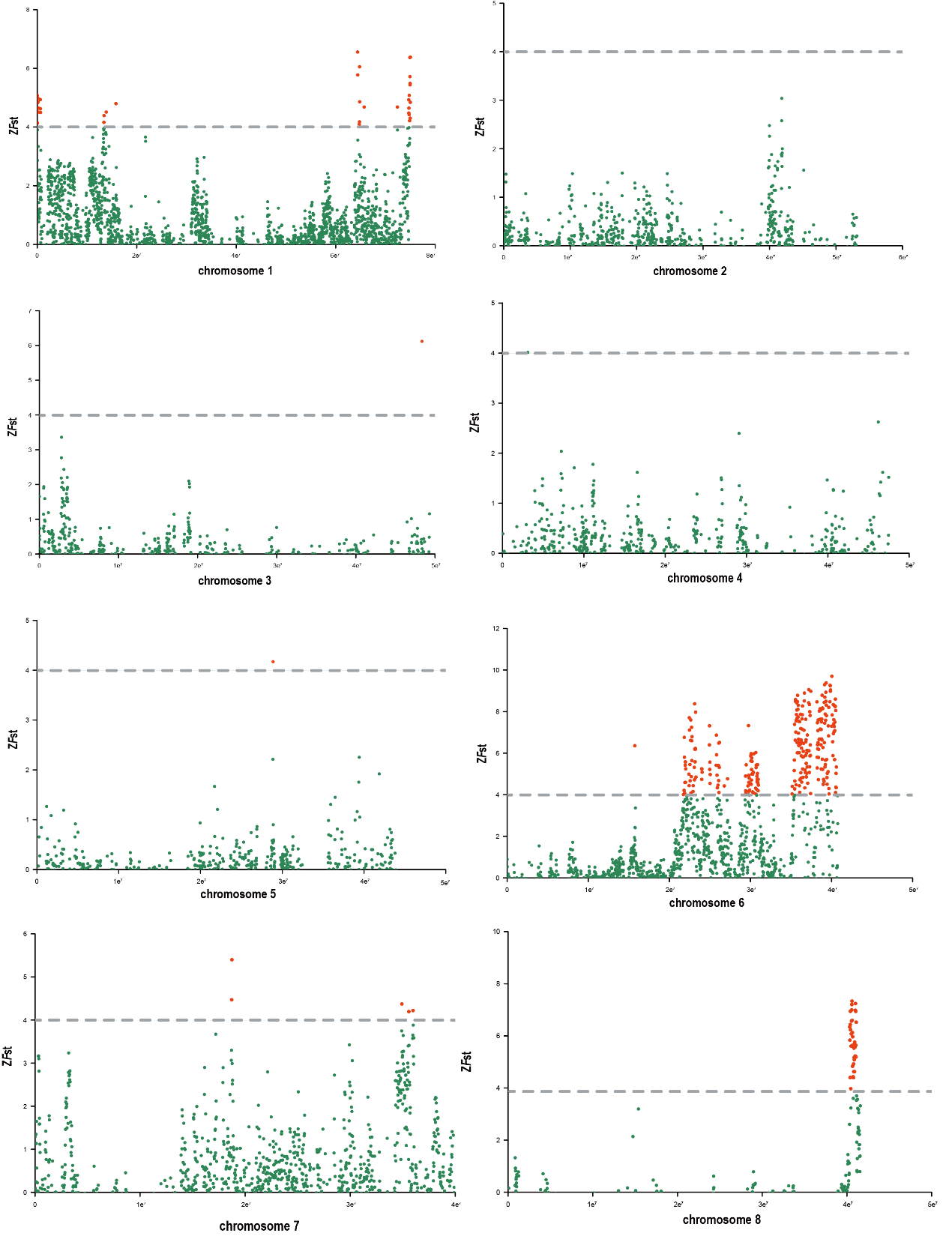


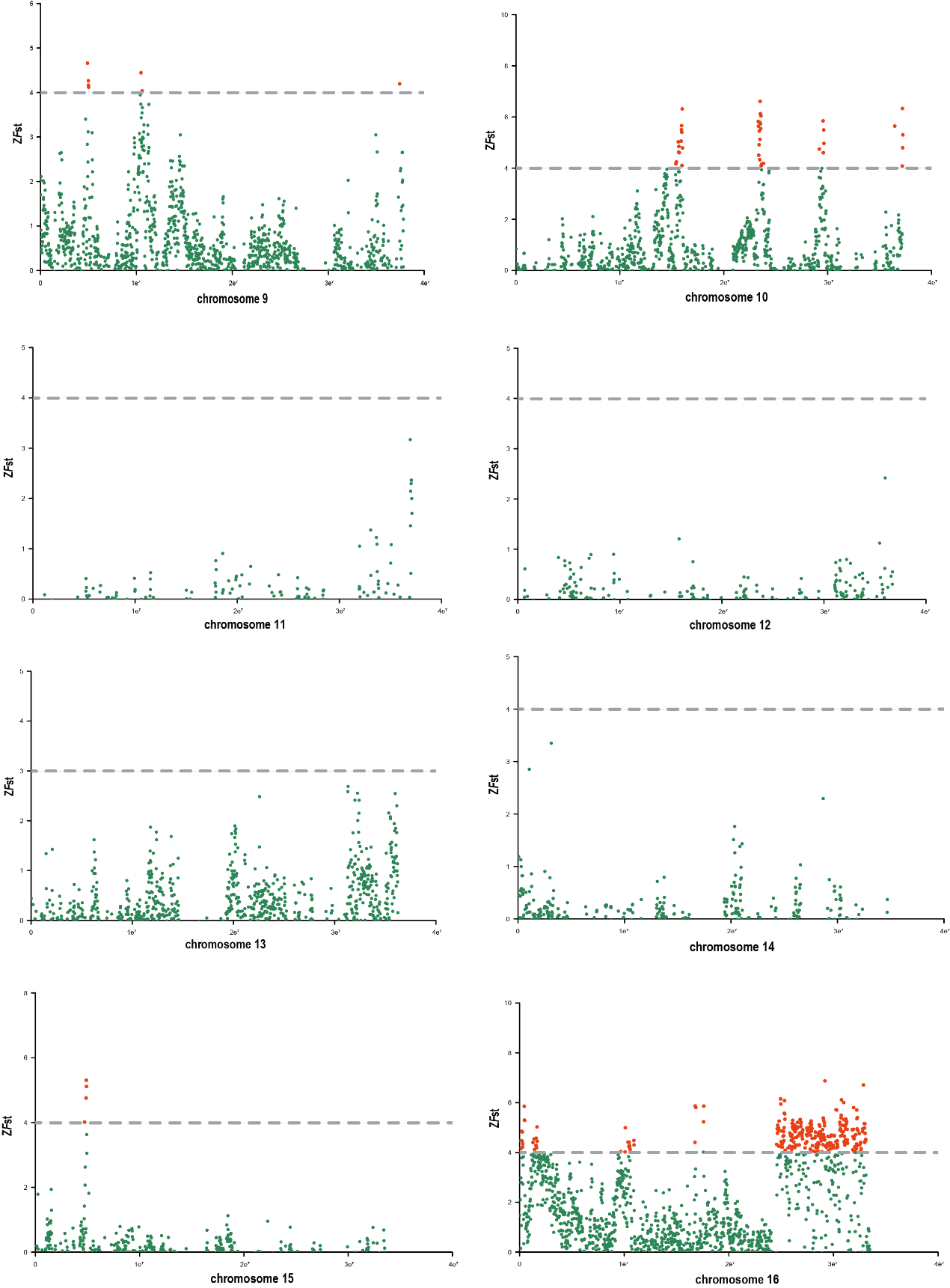


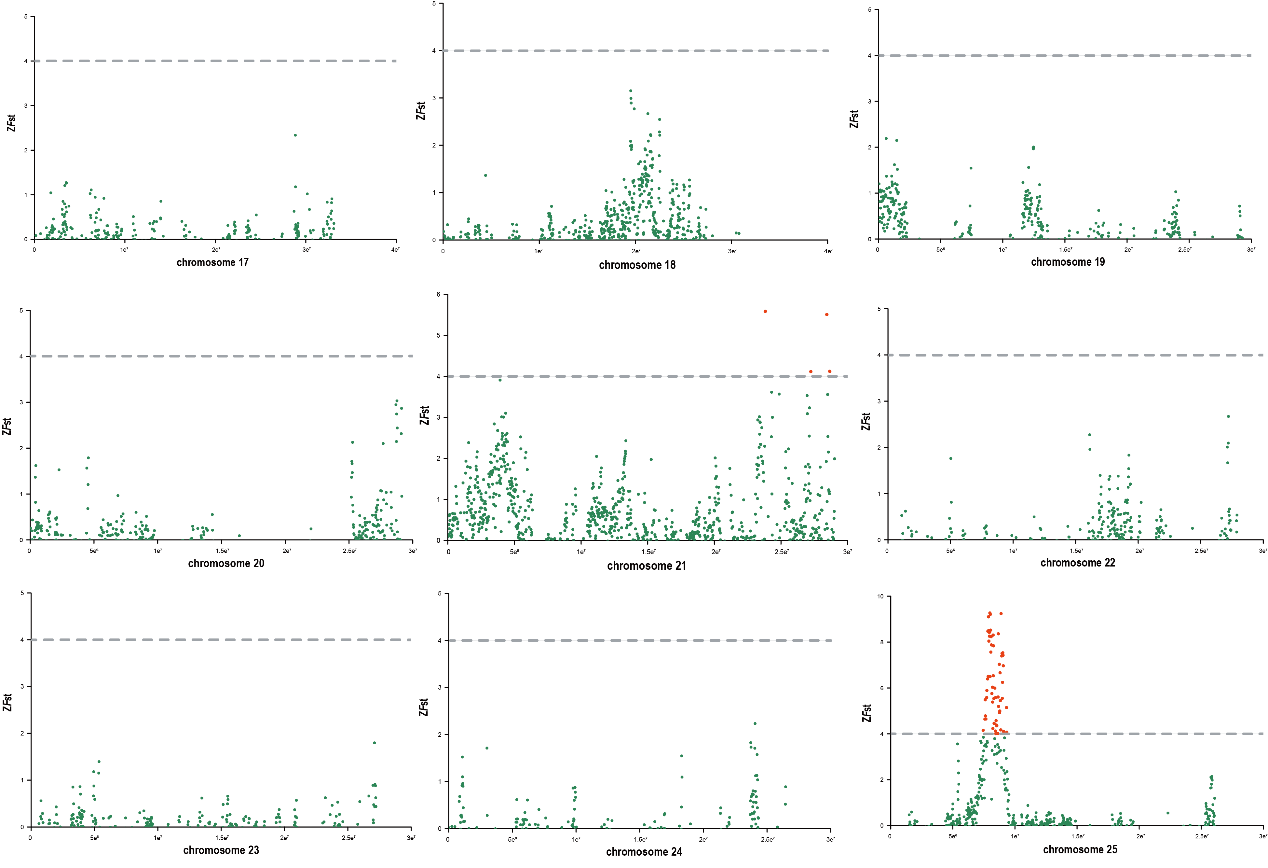


Supplementary Figure S14**.** Z-transformed pairwise genetic divergence (Z*F*st) scores calculated from 20 kb sliding windows across all chromosomes for *Gymnocypris eckloni scoliostomus* (GS) and *G. eckloni eckloni* (GE) comparison. Genomic islands are shown as red dots.


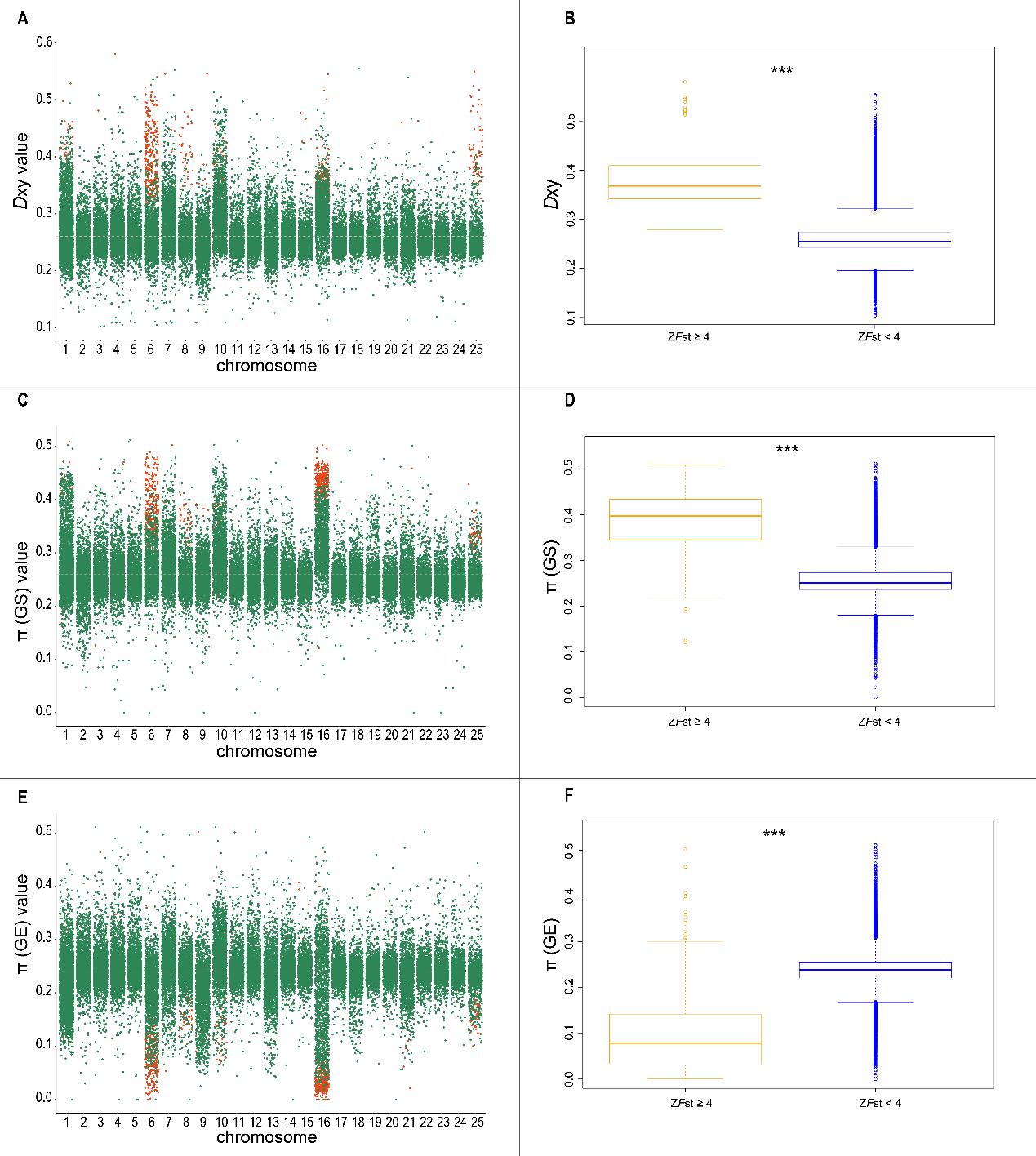


Supplementary Figure S15**.** Comparison of Nucleotide diversity (π) and absolute divergence (*D*xy) measure between genomic islands (Z*F*st ≥ 4) and the genomic background (Z*F*st < 4). Significant differences of Mann–Whitney U test are indicated with *** < 2.2×10^-16^.


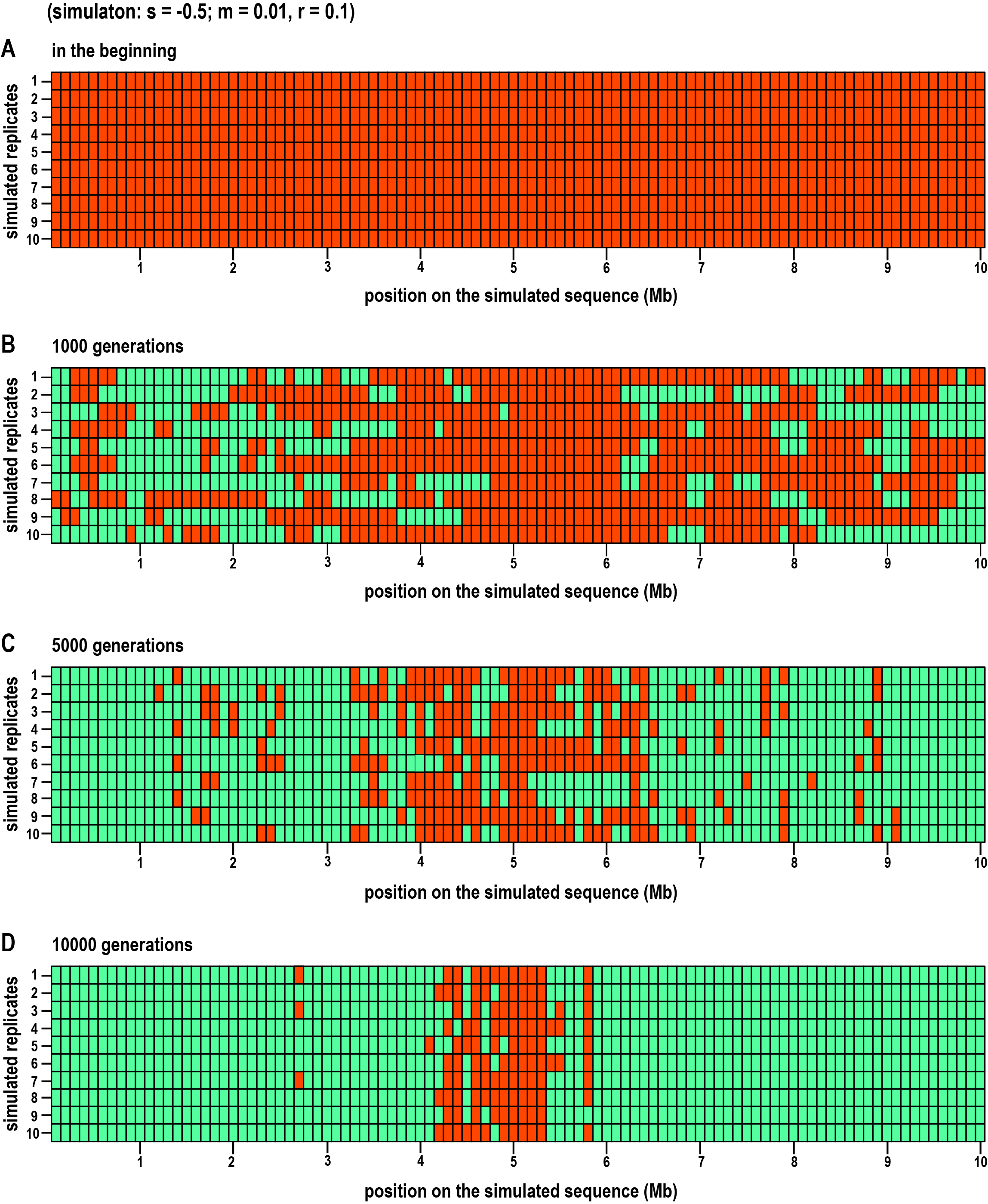


Supplementary Figure S16. Simulated introgressions in diploid 10 Mb genomes. Red and green represent sequences from two different species. (A). In the beginning, the sequences were in original sequence states. (B-D) Simulated results of 1,000, 5,000, 10,000, generations under strong selection (s = −0.5), weak migration rate (m = 0.01), recombination rate (r = 0.1 for per 10 Mb per generation). These results suggest that large genomic islands (≥ 100 kb) occur disproportionately in micro-parapatric speciation.


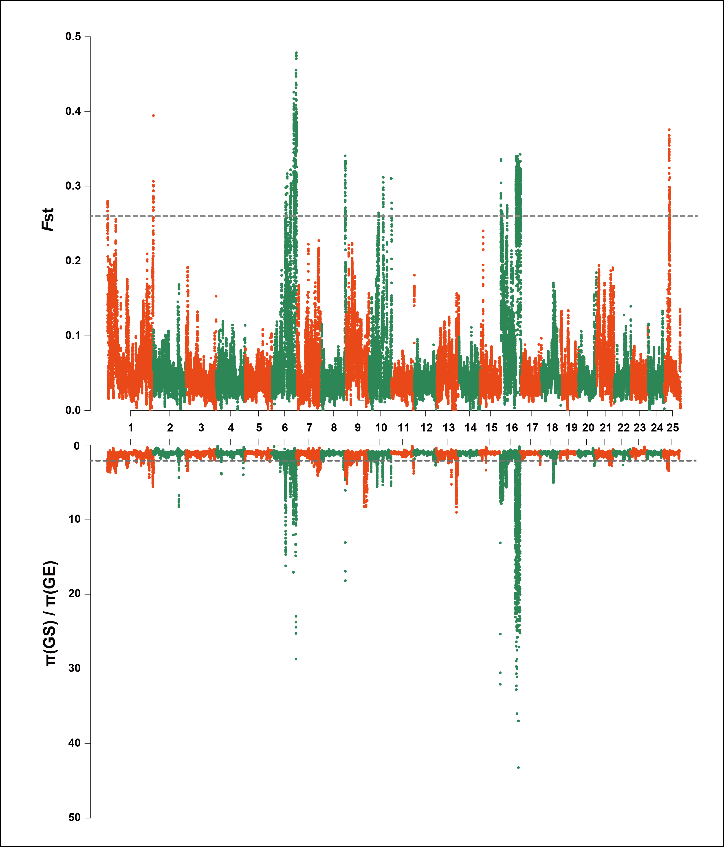


Supplementary Figure S17. Manhattan plots showing the selective sweep regions along the genome inferred from *F*st and π statistics. The grey dash lines indicate the top 5% π ratio threshold (π ratio = 2.07) and the top 1% *F*st threshold (*F*st = 0.26).

**
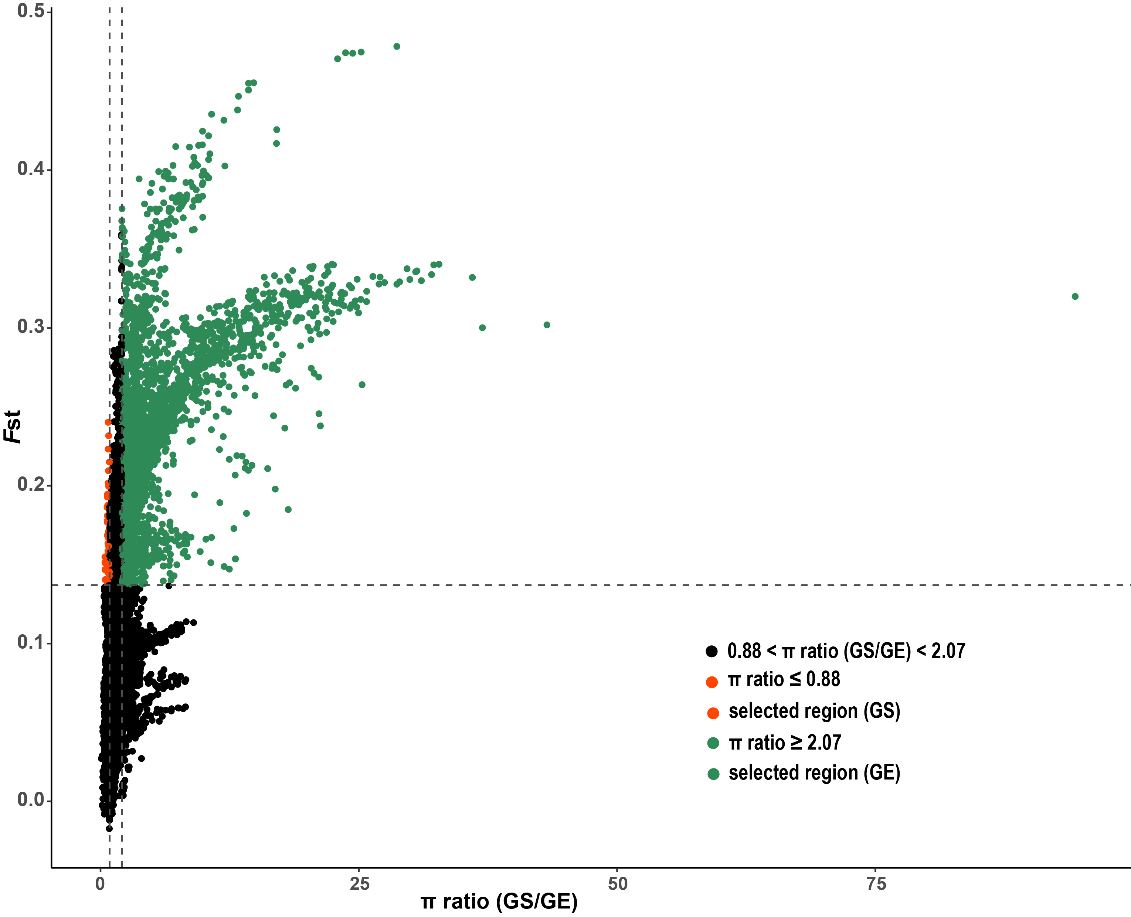
**

Supplementary Figure S18**.** Distribution of π ratio (GS/GE) and *F*st value. Green points represent the selective sweep regions of GE lineage (top 5% π ratio ≥ 2.07 and top 5% *F*st ≥ 0.137). Orange points represent the selective sweep regions of GS lineage (bottom 5% π ratio < 0.88 and top 5% *F*st ≥ 0.137). GS: *Gymnocypris eckloni scoliostomus*; GE: *Gymnocypris eckloni eckloni*.


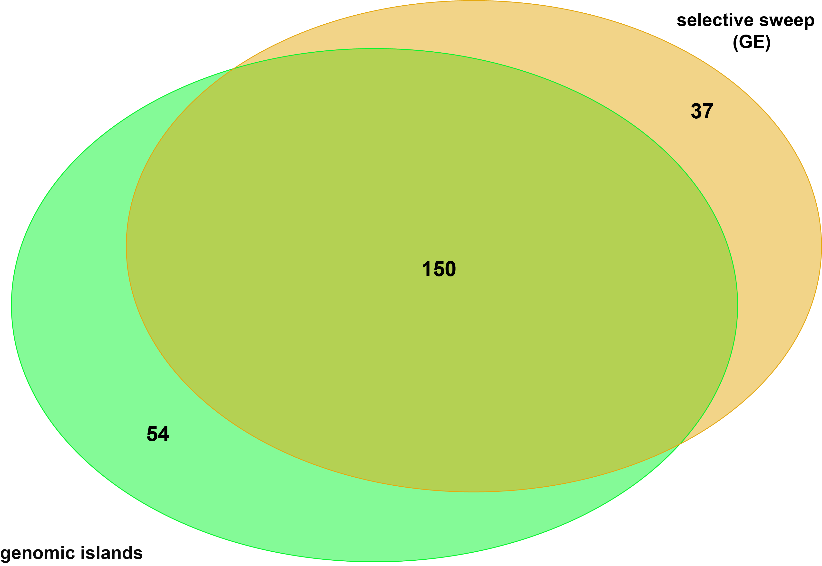


Supplementary Figure S19**.** Venn diagram of the number of genes from genomic islands and selective sweep regions of *Gymnocypris eckloni eckloni*.

# Supplementary Tables

Supplementary Table S1**.** Sequencing data for genome assembly of *Gymnocypris eckloni scoliostomus*.

| **Sequencing libraries** | **Insert size** | **Clean data (Gb)** | **Sequence coverage (x)** |
| --- | --- | --- | --- |
| Illumina reads | 250 bp | 85.85 | 90.56 |
| Nanopore reads | 20 kb | 137.83 | 145.38 |
| Hi-C reads | 250 bp | 217.24 | 229.16 |
| Total | - | 440.92 | 465.1 |

Supplementary Table S2**.** Estimated genome size with 17-mer analysis of *Gymnocypris eckloni scoliostomus*.

| **K-mer** | **K-mer**  **number** | **Peak**  **Depth** | **Genome Size**  **(bp)** | **Used Bases**  **(bp)** | **Used Reads** |
| --- | --- | --- | --- | --- | --- |
| 17 | 73,750,479,859 | 80 | 900,274,412 | 560,108,812 | 82,712,220,851 |

Supplementary Table S3. The statistics of the assembled contigs and scaffolds for *Gymnocypris eckloni scoliostomus* genome.

| **Term** | **Size**  **(bp)** | **Contig number** | **Size**  **(bp)** | **Scaffolds**  **number** |
| --- | --- | --- | --- | --- |
| N50 | 2,001,950 | 104 | 37,113,662 | 11 |
| N60 | 1,427,346 | 160 | 36,241,462 | 13 |
| N70 | 960,483 | 241 | 33,466,191 | 16 |
| N80 | 595,977 | 370 | 29,284,262 | 19 |
| N90 | 257,766 | 610 | 28,019,969 | 22 |
| Max length (bp) | 17,707,058 | 1 | 75,020,309 | 1 |
| Total size (bp) | 947,986,004 | 1292 | 948,103,204 | 120 |
| Total number (≥1 kp) | 947,986,004 | 1292 | 948,103,204 | 120 |
| Total number (≥2 kp) | 947,986,004 | 1292 | 948,103,204 | 120 |
| Total number (≥5 kb) | 947,986,004 | 1292 | 948,103,204 | 120 |

Supplementary Table S4. The summary of repetitive sequence for *Gymnocypris eckloni scoliostomus* genome.

|  | **ltr** | | **Repbase TEs** | | **TE proteins** | | **Trf** | | **RepeatModeler** | | **Combined TEs** | |
| --- | --- | --- | --- | --- | --- | --- | --- | --- | --- | --- | --- | --- |
| **Type** | **Length**  **(bp)** | **% in genome** | **Length**  **(bp)** | **% in genome** | **Length**  **(bp)** | **% in genome** | **Length**  **(bp)** | **% in genome** | **Length**  **(bp)** | **% in genome** | **Length**  **(bp)** | **% in genome** |
| DNA | 0 | 0 | 53,684,046 | 5.66 | 24,031,630 | 2.54 | 0 | 0 | 171,275,781 | 18.07 | 188,640,334 | 19.90 |
| LINE | 0 | 0 | 18,052,089 | 1.9 | 36,351,410 | 3.83 | 0 | 0 | 39,328,597 | 4.15 | 47,982,686 | 5.06 |
| SINE | 0 | 0 | 2,740,598 | 0.29 | 0 |  | 0 | 0 | 4,780,092 | 0.5 | 4,740,498 | 0.5 |
| LTR | 92,198,559 | 9.73 | 22,468,678 | 2.37 | 42,496,777 | 4.48 | 0 | 0 | 56,247,969 | 5.93 | 140,770,859 | 14.85 |
| Other | 0 | 0 | 9,257,873 | 0.97 | 155,844 | 0.02 | 46,750,188 | 4.93 | 7,481,408 | 1.3 | 48,862,789 | 8.67 |
| Unknown | 0 | 0 | 710,513 | 0.07 | 0 | 0 | 0 | 0 | 41,434,622 | 4.37 | 33,239,394 | 3.51 |
| Total | 92,198,559 | 9.73 | 2,263,399 | 11.26 | 103,035,661 | 10.87 | 46,750,188 | 4.93 | 320,548,469 | 34.32 | 464,236,560 | 52.49 |

Supplementary Table S5**.** General statistics of predicted protein-coding genes in *Gymnocypris eckloni scoliostomus* genome compared to other fish species*.* * indicates species is tetraploid.

| **Species** | **Genome**  **Size (Mb)** | **Number of gene** | **Average gene length (bp)** | **Average CDS length (bp)** | **Average exons per gene** | **Average exon length (bp)** | **Average introns per gene** | **Average intron length(bp)** |
| --- | --- | --- | --- | --- | --- | --- | --- | --- |
| *Schizothorax oconnori ** | 1940 | 43,731 | 13,389.89 | 1,508.89 | 8.85 | 170.49 | 7.94 | 1,513.45 |
| *Sinocyclocheilus grahami ** | 1753 | 45,778 | 16,259.73 | 1,587.57 | 9.25 | 171.70 | 6.30 | 1,779.42 |
| *Gymnocypris ecklonis scoliostomus ** | 905 | 24,194 | 19,483.05 | 1,740.08 | 10.59 | 164.38 | 9.59 | 1,805.96 |
| *Glyptosternum maculatum* | 704 | 22,846 | 17,782.28 | 1,762.04 | 10.31 | 170.85 | 6.65 | 1,720.14 |
| *Oryzias latipes* | 646 | 19,222 | 12,232.74 | 1,474.97 | 10.02 | 147.16 | 9.02 | 1,192.29 |
| *Danio rerio* | 1309 | 23,429 | 27,208.65 | 1,617.76 | 9.46 | 171.09 | 8.46 | 3,026.46 |
| *Oreochromis niloticus* | 1006 | 21,437 | 15,210.43 | 1,702.47 | 10.77 | 158.01 | 9.77 | 1,381.97 |
| *Triplophysa siluroides* | 583 | 25,406 | 10,311.35 | 1,536.79 | 8.96 | 171.52 | 7.96 | 1,102.36 |
| *Takifugu rubripes* | 384 | 18,506 | 7,710.91 | 1,659.52 | 11.08 | 149.77 | 10.08 | 600.32 |

Supplementary Table S6. BUSCO scores of the assembled *Gymnocypris eckloni scoliostomus* genome.

|  | **Count** | **Ratio** |
| --- | --- | --- |
| Complete BUSCOs | 4124 | 90% |
| Complete and single-copy BUSCOs | 3677 | 80.2% |
| Complete and duplicated BUSCOs | 447 | 9.8% |
| Fragmented BUSCOs | 228 | 5.0% |
| Missing BUSCOs | 232 | 5.0% |
| Total BUSCO groups searched | 4584 | - |

Supplementary Table S7**.** Statistics of reads mapping and coverage of 46 *Gymnocypris* species used in population genomics.

| **Group** | **Sample**  **ID** | **Clean reads**  **(Gb)** | | **Coverage**  **(x)** | **Mapping rate**  **(%)** |
| --- | --- | --- | --- | --- | --- |
| *Gymnocypris eckloni scoliostomus* | R14 | | 19.19 | 20.25 | 99.00 |
|  | R15 | | 18.81 | 19.84 | 99.17 |
|  | R16 | | 21.64 | 22.82 | 98.99 |
|  | R17 | | 21.33 | 22.50 | 98.87 |
|  | R18 | | 17.15 | 18.01 | 98.98 |
|  | R19 | | 18.13 | 19.12 | 99.00 |
|  | R21 | | 16.69 | 17.61 | 98.97 |
|  | R23 | | 22.82 | 24.08 | 98.76 |
|  | R25 | | 20.07 | 21.17 | 99.05 |
|  | R26 | | 17.34 | 18.29 | 98.98 |
|  | R27 | | 18.08 | 19.07 | 98.91 |
|  | R28 | | 18.94 | 19.98 | 99.02 |
|  | R29 | | 21.15 | 22.31 | 99.03 |
|  | R33 | | 15.43 | 16.27 | 98.96 |
|  | R34 | | 15.20 | 16.03 | 99.17 |
|  | R35 | | 16.19 | 17.08 | 99.09 |
|  | R36 | | 16.49 | 17.40 | 98.95 |
|  | R37 | | 16.61 | 17.53 | 99.03 |
|  | R39 | | 18.40 | 19.41 | 99.03 |
|  | R40 | | 17.06 | 17.99 | 99.05 |
|  | R41 | | 16.93 | 17.86 | 99.14 |
|  | R42 | | 17.42 | 18.37 | 99.13 |
|  | R50 | | 17.55 | 18.51 | 99.20 |
| *Gymnocypris eckloni eckloni* | R20 | | 19.05 | 20.09 | 99.03 |
|  | R22 | | 19.40 | 20.47 | 99.19 |
|  | R24 | | 14.94 | 15.76 | 98.92 |
|  | R30 | | 19.67 | 20.75 | 99.00 |
|  | R31 | | 17.65 | 18.62 | 98.92 |
|  | R32 | | 18.65 | 19.67 | 98.98 |
| **Group** | **Sample**  **ID** | | **Clean reads**  **(Gb)** | **Coverage**  **(x)** | **Mapping rate**  **(%)** |
| *Gymnocypris eckloni eckloni* | R38 | | 18.99 | 20.03 | 99.00 |
|  | R43 | | 17.34 | 18.29 | 99.04 |
|  | R44 | | 17.17 | 18.12 | 99.10 |
|  | R45 | | 17.18 | 18.12 | 99.14 |
|  | R46 | | 17.72 | 18.69 | 99.06 |
|  | R47 | | 18.77 | 19.80 | 99.13 |
|  | R48 | | 16.97 | 17.90 | 99.12 |
|  | R49 | | 17.60 | 18.57 | 99.16 |
|  | R51 | | 18.21 | 19.21 | 99.14 |
|  | R52 | | 17.28 | 18.23 | 99.11 |
|  | R53 | | 19.39 | 20.45 | 99.14 |
|  | R54 | | 18.61 | 19.64 | 99.06 |
|  | R55 | | 19.24 | 20.30 | 99.08 |
|  | R56 | | 20.16 | 21.27 | 99.12 |
|  | R57 | | 15.43 | 16.27 | 99.13 |
|  | R58 | | 16.20 | 17.09 | 98.93 |
|  | R59 | | 20.62 | 21.75 | 99.05 |

Supplementary Table S8**.** Population demographic parameters of *Gymnocypris eckloni*

*Scoliostomus* and *G. eckloni eckloni*.

| **Populations** | **Nucleotide diversity (π)** | **Tajima’s *D*** | **LD mean** *r*^2^ |
| --- | --- | --- | --- |
| *Gymnocypris eckloni scoliostomus* | 3.14×10^−3^ (95%CI:3.14×10^-3^-3.13×10^-3^) | 1.10 (95%CI:1.09-1.11) | 0.079 |
| *Gymnocypris eckloni eckloni* | 2.87×10^−3^ (95%CI:2.88×10^-3^- 2.86×10^-3^) | 1.05 (95%CI:1.05-1.07) | 0.086 |

Supplementary Table S9**.** CV error values for structure analysis (K=2−5).

| **Structure** | **CV error** |
| --- | --- |
| K=2 | 0.45172 |
| K=3 | 0.53779 |
| K=4 | 0.60304 |
| K=5 | 0.68144 |

Supplementary Table S10**.** Gene flow model estimation using Fastsmcoal2 and AIC.

| **Models** | **MaxEstLhood** | **N of estimated params** | **AIC** | **∆AIC** |
| --- | --- | --- | --- | --- |
| different gene flow | -25292709.912 | 9 | 116477251.609588 | 805359.96 |
| recent gene flow | -25482287.225 | 7 | 117350283.399355 | 994937.27 |
| early gene flow | -25491158.113 | 7 | 117391135.348296 | 1003808.16 |
| constant gene flow | -25491308.930 | 5 | 117391827.886248 | 1003958.97 |
| no gene flow | -25781878.556 | 4 | 118729948.464857 | 1294528.60 |

Supplementary Table S11**.** Comparison of population genomic parameters (the mean ± standard deviation values) of genomic islands with the rest of the genomic regions for all pairwise comparisons by Mann-Whitney U test. *D*xy: absolute divergence; π: nucleotide diversity; *ρ*: recombination rate.

| **Parameters** | | **Lineages** | | **Genomic islands** | **Genomic background** | ***P*-value** |
| --- | --- | --- | --- | --- | --- | --- |
| *D*xy | GS/GE | | | 0.3802±0.0525 | 0.2620±0.0355 | 2.2×10^-16^ |
| π | | | GS | 0.3886±0.0572 | 0.2591±0.0402 | 2.2×10^-16^ |
|  | | | GE | 0.0952±0.074 | 0.2369±0.0441 | 2.2×10^-16^ |
| *ρ* | | | GS | 78.6330 ±50.247 | 320.5216 ± 132.358 | 2.2×10^-16^ |
|  | | | GE | 75.9370 ± 71.108 | 292.1777 ± 128.411 | 2.2×10^-16^ |

Supplementary Table S12**.** GO enrichment analysis for genes of genomic islands.

| **ID** | **GO name** | **Ontology** | **Count** | ***P*-value** |
| --- | --- | --- | --- | --- |
| GO:0004984 | olfactory receptor activity | MF | 6 | 2.23×10^-5^ |
| GO:0005249 | voltage-gated potassium channel activity | MF | 5 | 2.67×10^-3^ |
| GO:0007186 | G-protein coupled receptor signaling pathway | BP | 12 | 1.05×10^-2^ |
| GO:0050896 | response to stimulus | BP | 26 | 1.86×10^-2^ |
| GO:0034703 | cation channel complex | CC | 4 | 1.38×10^-2^ |
| GO:0004871 | signal transducer activity | MF | 16 | 1.6×10^-2^ |
| GO:0003924 | GTPase activity | MF | 6 | 4.19×10^-2^ |
| GO:0006470 | protein dephosphorylation | BP | 4 | 2.53×10^-2^ |
| GO:0004721 | phosphoprotein phosphatase activity | MF | 4 | 4.68×10^-2^ |

Supplementary Table S13**.** GO enrichment analysis for genes of genomic islands with a significant *P*-value.

| **ID** | **GO name** | **Chromosome** | **Gene** |
| --- | --- | --- | --- |
| GO:0004984 | olfactory receptor activity | 6.638 | *or52k2* |
|  |  | 6.639 | *or52k1* |
|  |  | 6.64 | *or52k1* |
|  |  | 6.66 | *or3a3* |
|  |  | 6.663 | *or52k1* |
|  |  | 6.669 | *or52k1* |
| GO:0005249 | voltage-gated potassium channel activity | 6.974 | *kcna1* |
|  |  | 6.976 | *kcna1* |
|  |  | 6.977 | *tsha2* |
|  |  | 16.482 | *kcng2* |
|  |  | 16.543 | *kcnh1* |
| GO:0007186 | G-protein coupled receptor signaling pathway | 4.74 | *pcyt2* |
|  |  | 6.638 | *or52k2* |
|  |  | 6.639 | *or52k1* |
|  |  | 6.64 | *or52k1* |
|  |  | 6.66 | *or3a3* |
|  |  | 6.663 | *or52k1* |
|  |  | 6.669 | *or52k2* |
|  |  | 6.76 | *p2ry13* |
|  |  | 10.537 | *oprd1* |
|  |  | 16.288 | *adgrb2* |
|  |  | 16.494 | *mc5r* |
|  |  | 25.114 | *gnas* |
| GO:0050896 | response to stimulus | 4.74 | *pcyt2* |
|  |  | 6.615 | *pik3cb* |
|  |  | 6.638 | *or52k2* |
|  |  | 6.639 | *or52k1* |
|  |  | 6.64 | *or52k1* |
|  |  | 6.66 | *or3a3* |

| **ID** | **GO name** | **Chromosome** | **Gene** |
| --- | --- | --- | --- |
| GO:0050896 | response to stimulus | 6.663 | *or52k1* |
|  |  | 6.666 | *defb3* |
|  |  | 6.669 | *or52k1* |
|  |  | 6.76 | *p2py13* |
|  |  | 6.948 | *tlr13* |
|  |  | 7.808 | *3arhgef3* |
|  |  | 7.481 | *sbno2* |
|  |  | 9.116 | *fgfrla* |
|  |  | 10.537 | *oprd1* |
|  |  | 16.288 | *adgrb2* |
|  |  | 16.48 | *ldlrad4* |
|  |  | 16.489 | *smad4* |
|  |  | 16.494 | *mc5r* |
|  |  | 16.5 | *ppp1r2b* |
|  |  | 16.546 | *traf5* |
|  |  | 16.563 | *arhgap19* |
|  |  | 16.565 | *efemp1* |
|  |  | 21.617 | *irf7* |
|  |  | 25.114 | *gnas* |
|  |  | 25.119 | *gli3* |
| GO:0034703 | cation channel complex | 6.974 | *kcna1* |
|  |  | 6.976 | *kcna1* |
|  |  | 6.977 | *tsha2* |
|  |  | 16.482 | *kcng2* |
| GO:0004871 | signal transducer activity | 6.638 | *or52k2* |
|  |  | 6.639 | *or52k1* |
|  |  | 6.64 | *or52k1* |
|  |  | 6.66 | *or3a3* |
|  |  | 6.663 | *or52k1* |
|  |  | 6.669 | *or52k1* |
|  |  | 6.76 | *p2ry13* |
|  |  | 6.948 | *tlr13* |

| **ID** | **GO name** | **Chromosome** | **Gene** |
| --- | --- | --- | --- |
| GO:0004871 | signal transducer activity | 8.1019 | *grik2* |
|  |  | 9.116 | *fgfr1a* |
|  |  | 9.296 | *gf2a2* |
|  |  | 10.537 | *oprd1* |
|  |  | 16.288 | *adgrb2* |
|  |  | 16.494 | *mc5r* |
|  |  | 16.565 | *efemp1* |
|  |  | 16.572 | *htr3a* |
| GO:0003924 | GTPase activity | 4.74 | *pcyt2* |
|  |  | 6.919 | *rab19* |
|  |  | 6.973 | *trim24* |
|  |  | 16.489 | *smad4* |
|  |  | 21.617 | *irf7* |
|  |  | 25.114 | *gnas* |
| GO:0006470 | protein dephosphorylation | 6.966 | *dusp29* |
|  |  | 6.967 | *dusp29* |
|  |  | 16.478 | *ptpn2* |
|  |  | 16.6 | *acap2* |
| GO:0004721 | phosphoprotein phosphatase activity | 6.966 | *dusp29* |
|  |  | 6.967 | *dusp29* |
|  |  | 16.171 | *ptpn2* |
|  |  | 16.478 | *eya3* |

Supplementary Table S14**.** KEGG enrichment analysis for genes of genomic islands.

| Pathway ID | KEGG name | Genes |
| --- | --- | --- |
| ko04740 | olfactory transduction | *or52k1*, *or52k2*, *ncaldb*, *gnal*, *or3a3* |
| ko04080 | neuroactive ligand-receptor interaction | *oprd1*, *grik2*, *mc5r* |
| ko04024 | cAMP signaling pathway | *phkg1*, *gil3* |
| ko04020 | calcium signaling pathway | *phkg1*, *gnas* |
| ko04015 | Rap1 signaling pathway | *pfn2*, *pgfr1a* |

Supplementary Table S15**.** GO enrichment analysis for sweep selective genes of *Gymnocypris eckloni eckloni*. The selective genes obtained from *F*st and π method.

| **ID** | **GO name** | **Ontology** | **Count** | ***P*-value** |
| --- | --- | --- | --- | --- |
| GO:0006470 | protein dephosphorylation activity | BP | 7 | 1.11×10^-5^ |
| GO:0004721 | phosphoprotein phosphatase activity | MF | 6 | 3.51×10^-4^ |
| GO:0005249 | voltage-gated potassium channel activity | MF | 5 | 1.68×10^-3^ |
| GO:0004984 | olfactory receptor activity | MF | 3 | 1.34×10^-2^ |
| GO:0005216 | ion channel activity | MF | 7 | 2.94×10^-2^ |

Supplementary Table S16**.** GO enrichment analysis for sweep selective genes of *Gymnocypris eckloni eckloni* with a significant *P*-value. The selective genes obtained from *F*st and π method.

| **ID** | **GO name** | **Chromosome** | **Gene** |
| --- | --- | --- | --- |
| GO:0006470 | protein dephosphorylation activity | 6.961 | *ptprb* |
|  |  | 6.966 | *dusp29* |
|  |  | 6.967 | *dusp29* |
|  |  | 6.968 | *ptpn5* |
|  |  | 16.475 | *ctdp1* |
|  |  | 16.478 | *ptpn2* |
|  |  | 16.5 | *ppp1r2* |
| GO:0004721 | phosphoprotein phosphatase activity | 6.961 | *ptprb* |
|  |  | 6.966 | *dusp29* |
|  |  | 6.967 | *dusp29* |
|  |  | 6.968 | *ptpn5* |
|  |  | 16.475 | *ctdp1* |
|  |  | 16.478 | *ptpn2* |
| GO:0005249 | voltage-gated potassium channel activity | 6.974 | *tsha2* |
|  |  | 6.976 | *kcna1* |
|  |  | 6.977 | *kcna1* |
|  |  | 16.482 | *kcng2* |
|  |  | 16.543 | *kcnh1* |
| GO:0004984 | olfactory receptor activity | 6.638 | *or52k2* |
|  |  | 6.639 | *or52k1* |
|  |  | 6.64 | *or52k1* |
| GO:0005216 | ion channel activity | 6.974 | *tsha2* |
|  |  | 6.976 | *grik2* |
|  |  | 6.977 | *kcna1* |
|  |  | 8.1019 | *kcng2* |
|  |  | 16.482 | *htr3a* |
|  |  | 16.543 | *kcnh1* |
|  |  | 16.572 | *kcna1* |

Supplementary Table S17**.** KEGG enrichment analysis for sweep selective genes of *Gymnocypris eckloni eckloni*. The selective genes obtained from *F*st and π method.

| **Pathway ID** | **KEGG name** | **Genes** |
| --- | --- | --- |
| ko04740 | olfactory transduction | *Or5rk1*, *or52k2*, *ncaldb*, *gnal* |
| ko04080 | neuroactive ligand-receptor interaction | *oprd1*, *grik2*, *mc5r*, *endr* |
| ko04020 | calcium signaling pathway | *phkg1*, *gnas* |
| ko04022 | cGMP - PKG signaling pathway | *srf*, *oprd1* |

**Supplementary Table S18.** GO enrichment analysis for sweep selective genes of *Gymnocypris eckloni eckloni.* The selective genes obtained from XP-CLR method.

| **ID** | **GO name** | **Ontology** | **Count** | ***P*-value** |
| --- | --- | --- | --- | --- |
| GO:0004984 | olfactory receptor activity | MF | 4 | 4.86×10^-3^ |
| GO:0050896 | response to stimulus | BP | 35 | 9.24×10^-3^ |
| GO:0043169 | cation binding | MF | 24 | 3.26×10^-2^ |
| GO:0006811 | ion transport | BP | 12 | 4.15×10^-2^ |

**Supplementary Table S19.** KEGG enrichment analysis for sweep selective genes of *Gymnocypris eckloni eckloni.* The selective genes obtained from XP-CLR method.

| Pathway ID | KEGG name | Genes |
| --- | --- | --- |
| ko04740 | olfactory transduction | *or52k1*, *olfr7*, *gnal*, |
| ko04080 | neuroactive ligand-receptor interaction | *taar6*, *grik2*, *edn2*, *p2ry10*, *uts2r*, *hrh3*, *grin2a* |
| ko04020 | calcium signaling pathway | *cacna1g*, *gnal*, *plcd3*, *grin2a* |
| ko04015 | Rap1 signaling pathway | *lcp2, ralgds, rapgef2*, *src*, *grin2a* |

**Supplementary Table S20.** GO enrichment analysis for the shared selective sweep genes of *Gymnocypris eckloni eckloni*. The shared selective genes obtained from XP-CLR and *F*st and π methods.

| **ID** | **GO name** | **Ontology** | **Count** | ***P*-value** |
| --- | --- | --- | --- | --- |
| GO:0042221 | response to chemical | BP | 6 | 5.64×10^-3^ |
| GO:0004721 | response to stimulus | BP | 23 | 9.89×10^-3^ |
| GO:0007165 | signal transduction | BP | 21 | 5.96×10^-3^ |
| GO:0004984 | olfactory receptor activity | MF | 2 | 4.84×10^-2^ |

# Supplementary reference

1. Luo R, Liu B and Xie Y *et al*. SOAPdenovo2: an empirically improved memory-efficient short-read de novo assembler. *GigaScience* 2012; **1**: 2047–217X–1–18.
2. Koren S, Walenz BP and Berlin K *et al*. Canu: Scalable and accurate long-read assembly via adaptive k-mer weighting and repeat separation. *Genome Res* 2017; **27**: 722–36.
3. Kolmogorov M, Yuan J and Lin Y *et al*. Assembly of long, error-prone reads using repeat graphs. *Nat Biotechnol* 2019; **37**: 540–46.
4. Vaser R, Sovic I and Nagarajan N *et al*. Fast and accurate de novo genome assembly from long uncorrected reads. *Genome* *Res* 2017; **27**: 737–46.
5. Walker BJ, Abeel T and Shea T *et al*. Pilon: An integrated tool for comprehensive microbial variant detection and genome assembly improvement. *PLoS One* 2014; **9**: e112963.
6. Servant N, Varoquaux N and Lajoie BR *et al*. HiC-Pro: an optimized and flexible pipeline for Hi-C data processing. *Genome Bio* 2015; **16**: 1–11.
7. Langmead B, Trapnell C and Pop M *et al*. Ultrafast and memory-efficient alignment of short DNA sequences to the human genome. *Genome Bio* 2009; **10**: 1–10.
8. Burton JN, Adey A and Patwardhan RP *et al*. Chromosome-scale scaffolding of de novo genome assemblies based on chromatin interactions. *Nat* *Biotechnology* 2013; **31**: 1119–25.
9. Tarailo-Graovac M and Chen N. Using RepeatMasker to identify repetitive elements in 749 genomic sequences. *Curr Protoc Bioinformatics* 2009; **25**: 4–10.
10. Jurka J, Kapitonov VV and Pavlicek A *et al*. Repbase update, a database of eukaryotic repetitive elements. *Cytogenetic Genome Res* 2005; **110**: 462–67.
11. Benson G. Tandem repeats finder: a program to analyze DNA sequences. *Nucleic Acids Res* 1999; **27**: 573–80.
12. Stanke M, Diekhans M and Baertsch R *et al*. Using native and syntenically mapped cDNA alignments to improve de novo gene finding. *Bioinformatics* 2008; **24**: 637–44.
13. Alioto T, Blanco E and Parra G *et al*. Using geneid to identify genes. *Curr Protoc Bioinformatics* 2018; **64**: e56.
14. Majoros WH, Pertea M and Salzberg SL. TigrScan and GlimmerHMM: two open source ab initio eukaryotic gene-finders. *Bioinformatics* 2004; **20**: 2878–79.
15. Korf I. Gene finding in novel genomes. *BMC Bioinformatics* 2004; **5**: 1–9.
16. Altschul SF, Gish W and Miller W *et al*. Basic local alignment search tool. *J Mol Biol* 1990; **215**: 403–10.
17. Birney E, Clamp M and Durbin R. GeneWise and genomewise. *Genome Res* 2004; **14**:988–95.
18. Kent WJ. BLAT-the BLAST-like alignment tool. *Genome* *Res* 2002; **12**: 656–64.
19. Haas BJ, Delcher AL and Mount SM *et al*. Improving the Arabidopsis genome annotation using maximal transcript alignment assemblies. *Nucleic Acids Res* 2003; **31**: 5654–66.
20. Haas BJ, Salzberg SL and Zhu W. Automated eukaryotic gene structure annotation using evidencemodeler and the program to assemble spliced alignments. *Genome Biol* 2008; **9**: R7.
21. Li H. Aligning sequence reads, clone sequences and assembly contigs with BWA-MEM. Preprint at https://arxiv.org/abs/1303.3997 (2013).
22. Li H, Handsaker B and Wysoker A *et al*. The sequence alinment/map format and SAMtools. *Bioinformatrics* 2009; **25**: 2078–79.
23. Emms DM and Kelly S. OrthoFinder: solving fundamental biases in whole genome comparisons dramatically improves orthogroup inference accuracy. *Genome biol* 2015; **16**: 1–14.
24. Katoh K and Standley DM. MAFFT multiple sequence alignment software version 7: improvements in performance and usability. *Mol Biol Evol* 2013; **30**: 772–80.
25. Minh BQ, Schmidt HA and Chernomor O *et al*. IQ-TREE 2: new models and efficient methods for phylogenetic inference in the genomic era. *Mol Biol Evol* 2020; **37**: 1530–34 (2020).
26. Yang Z. PAML 4: phylogenetic analysis by maximum likelihood. *Mol Biol Evol* 2007; **24**: 1586–91.
27. Saitou N and Nei M. The neighbor-joining method: A new method for reconstructing phylogenetic trees. *Mol Biol Evol* 1987; **4**: 406–25.
28. Purcell S, Neale B and Todd-Brown K *et al*. PLINK: a tool set for whole-genome association and population-based linkage analyses. *AM J Hum Genet* 2007; **81**: 559–75.
29. Zhang C, Dong SS and Xu JY *et al*. PopLDdecay: a fast and effective tool for linkage disequilibrium decay analysis based on variant call format files. *Bioinformatics* 2019; **35**: 1786–88.
30. Gao F, Ming C and Hu W. New software for the fast estimation of population recombination rates (FastEPRR) in the genomic era. *G3-Genes Genom Genet* 2016; **6**: 1563–71.
31. Karlsson EK, Baranowska I and Wade CM *et al*. Efficient mapping of mendelian traits in dogs through genome-wide association. *Nat Gent* 2007; **39**: 1321–28.
32. Browning SR and Browning BL. Rapid and accurate haplotype phasing and missing-data inference for whole-genome association studies by use of localized haplotype clustering. *Am J Hum Genet* 2007; **81**: 1084–97.
